# Adjudin improves beta cell maturation, hepatic glucose uptake and glucose homeostasis

**DOI:** 10.1007/s00125-023-06020-4

**Published:** 2023-10-16

**Authors:** Lipeng Ren, Jérémie Charbord, Lianhe Chu, Aurino M. Kemas, Maria Bertuzzi, Jiarui Mi, Chen Xing, Volker M. Lauschke, Olov Andersson

**Affiliations:** 1 Department of Cell and Molecular Biology, Karolinska Institutet, Stockholm, Sweden; 2 Department of Physiology and Pharmacology, Karolinska Institutet, Stockholm, Sweden; 3 Department of Neuroscience, Karolinska Institutet, Stockholm, Sweden; 4 Dr Margarete Fischer-Bosch Institute of Clinical Pharmacology, Stuttgart, Germany; 5 Tübingen University, Tübingen, Germany

**Keywords:** Adjudin, Glucose uptake in liver, Insulin independence, Pancreatic beta cell maturation, Type 2 diabetes

## Abstract

**Aims/hypothesis:**

Recovering functional beta cell mass is a promising approach for future diabetes therapies. The aim of the present study is to investigate the effects of adjudin, a small molecule identified in a beta cell screen using zebrafish, on pancreatic beta cells and diabetes conditions in mice and human spheroids.

**Methods:**

In zebrafish, insulin expression was examined by bioluminescence and quantitative real-time PCR (qPCR), glucose levels were examined by direct measurements and distribution using a fluorescent glucose analogue, and calcium activity in beta cells was analysed by in vivo live imaging. Pancreatic islets of wild-type postnatal day 0 (P0) and 3-month-old (adult) mice, as well as adult *db/db* mice (i.e. BKS(D)-*Lepr*^*db*^/JOrlRj), were cultured in vitro and analysed by qPCR, glucose stimulated insulin secretion and whole mount staining. RNA-seq was performed for islets of P0 and *db/db* mice. For in vivo assessment, *db/db* mice were treated with adjudin and subjected to analysis of metabolic variables and islet cells. Glucose consumption was examined in primary human hepatocyte spheroids.

**Results:**

Adjudin treatment increased insulin expression and calcium response to glucose in beta cells and decreased glucose levels after beta cell ablation in zebrafish. Adjudin led to improved beta cell function, decreased beta cell proliferation and glucose responsive insulin secretion by decreasing basal insulin secretion in in vitro cultured newborn mouse islets. RNA-seq of P0 islets indicated that adjudin treatment resulted in increased glucose metabolism and mitochondrial function, as well as downstream signalling pathways involved in insulin secretion. In islets from *db/db* mice cultured in vitro, adjudin treatment strengthened beta cell identity and insulin secretion. RNA-seq of *db/db* islets indicated adjudin-upregulated genes associated with insulin secretion, membrane ion channel activity and exocytosis. Moreover, adjudin promoted glucose uptake in the liver of zebrafish in an insulin-independent manner, and similarly promoted glucose consumption in primary human hepatocyte spheroids with insulin resistance. In vivo studies using *db/db* mice revealed reduced nonfasting blood glucose, improved glucose tolerance and strengthened beta cell identity after adjudin treatment.

**Conclusions/interpretation:**

Adjudin promoted functional maturation of immature islets, improved function of dysfunctional islets, stimulated glucose uptake in liver and improved glucose homeostasis in *db/db* mice. Thus, the multifunctional drug adjudin, previously studied in various contexts and conditions, also shows promise in the management of diabetic states.

**Data availability:**

Raw and processed RNA-seq data for this study have been deposited in the Gene Expression Omnibus under accession number GSE235398 (https://www.ncbi.nlm.nih.gov/geo/query/acc.cgi?acc=GSE235398).

**Graphical Abstract:**

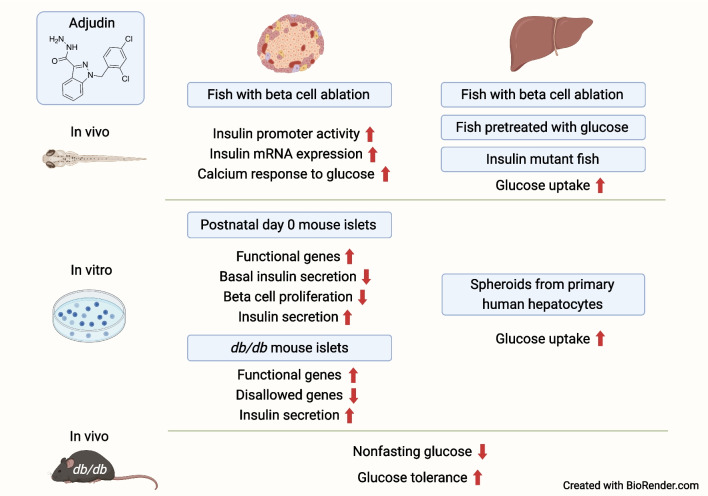

**Supplementary Information:**

The online version contains peer-reviewed but unedited supplementary material available at 10.1007/s00125-023-06020-4.


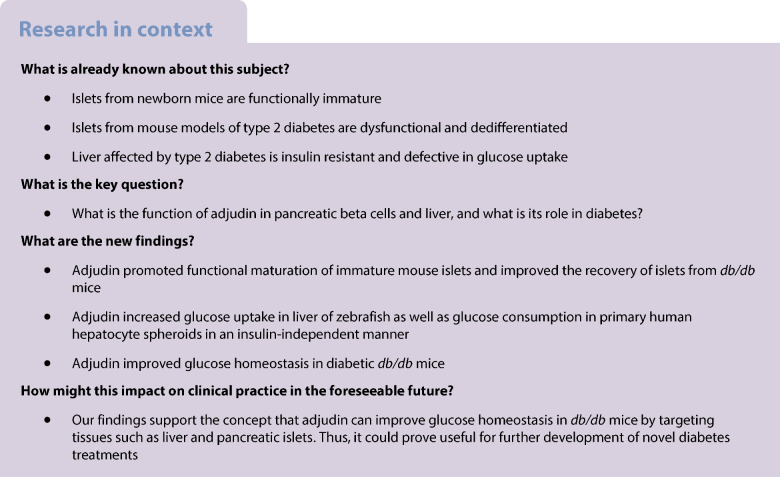


## Introduction

A precise control of insulin secretion is critical for maintenance of normoglycaemia. Insulin is exclusively produced and released by pancreatic beta cells, mainly via glucose stimulated insulin secretion (GSIS). In this process, glucose is transported into beta cells by glucose transporters, phosphorylated by glucokinase and converted to pyruvate through glycolysis [1]. Pyruvate enters the tricarboxylic acid (TCA) cycle in mitochondria and stimulates oxidative phosphorylation. The metabolism of glucose increases the ATP:ADP ratio and leads to closure of the ATP-sensitive potassium channel in membrane, resulting in cell depolarisation. In response to membrane depolarisation, voltage-gated calcium channels open and influx of calcium triggers insulin exocytosis [2]. In mice, glucose responsive insulin secretion develops in beta cells within 1–2 weeks after birth [3]. Maturation of beta cells is the process in which they acquire the capability to secrete insulin in response to elevated glucose and reduce basal insulin secretion. Adult or mature beta cells secrete little insulin in low glucose and exhibit robust insulin secretion in response to challenges of high glucose while neonatal or immature beta cells are known for their high basal insulin secretion, meaning that they have a low threshold for glucose stimulation and secrete high levels of insulin even in response to low glucose [3–5].

In type 2 diabetes, insulin resistance drives beta cells to secrete more insulin to meet the physiological requirements. Under such constant stress, beta cells become dysfunctional and eventually lose their identity. In vivo mouse and in vitro human islet studies suggested that beta cells are dedifferentiated in type 2 diabetes, as they lose expression of the typical beta cell transcription factors, including MafA (MAF bZIP transcription factor A), NKX6.1 (NK6 homeobox 1) and PDX1 (pancreatic and duodenal homeobox 1) [6–8]. As a result, islets from type 2 diabetes display impaired or blunted GSIS [7, 9, 10].

The liver is a crucial organ for glucose homeostasis since it disposes of approximately one-third of glucose postprandially [11]. The regulation of glucose homeostasis in liver involves increased hepatic glucose uptake primarily through activation of glycogen synthesis and decreased hepatic glucose production mediated by insulin [11, 12]. Hepatic glucose uptake is largely insulin independent as major glucose transporters in hepatocytes are not regulated by insulin; however, insulin can stimulate glucose uptake in liver partially through increasing intracellular glucose utilisation [13, 14]. In type 2 diabetes, both hepatic glucose uptake and suppression of hepatic glucose production are impaired [11, 12, 15], leading to uncontrolled hyperglycaemia.

Adjudin was identified in a screen for small molecules stimulating beta cell differentiation in zebrafish, i.e. via assessing effects on beta cell maturation by hits that increased insulin expression without increasing the number of beta cells. It is a multifunctional drug with known effects as a potential reversible male contraceptive, by inducing exfoliation of immature spermatids from seminiferous epithelium through disrupting the apical ectoplasmic specialisation, a testis-specific cell junction [16], and inhibiting sperm capacitation and fertilising capacity by blocking chloride channels [17]; an anti-cancerogenic through suppression of mitochondrial function and inhibition of proliferation [18]; an anti-neuroinflammation drug by inhibition of NF-κB [19]; and an anti-oxidative stress drug via activation of SIRT3 (sirtuin 3) [20]. The aim of present study is to investigate the role of adjudin in pancreatic beta cells and diabetes conditions in zebrafish, mice and human spheroids.

## Methods

### Zebrafish

All zebrafish experiments were conducted in accordance with the guidelines approved by Stockholms Djurförsöksetiska Nämnd. The following previously established lines were used: *Tg(ins:H2BGFP)*^*KI112*^, *Tg(ins:CFP-NTR)*^*s892*^, *ins*^*bns102*^, *Tg(ins:GCaMP6s;cryaa:RFP)*, abbreviated as *Tg(ins:GCaMP6s)* [21], and *Tg(ins:Flag-NTR)*^*s950*^. *Tg(ins:NLuc*;*cryaa:YFP)*^*KI120*^, abbreviated *Tg(ins:NLuc)*, was constructed by placing nanoluciferase downstream of the *ins* promoter by In-Fusion Cloning (639648, Takara Bio, Japan). *Tg(ins:H2BmCherry;cryaa:CFP)*^*KI147*^, abbreviated as *Tg(ins:H2BmCherry)*, was constructed by replacing the *loxp-mCherry-STOP-loxp-H2BGFP* cassette with H2BmCherry in the *Tg(ins:CSH;cryaa:CFP)* construct using In-Fusion Cloning (639648, Takara Bio).

### Mice

All mouse experiments were conducted in accordance with the guidelines approved by Stockholms Djurförsöksetiska Nämnd. Animals were housed in groups of maximum four in standardised cages with a 12 h light cycle and free access to regular chow diet and water.

For islet isolation and culture, 3–4-month-old male mice were used for isolation of adult islets. Postnatal day 0 (P0), P4 and P7 mice, regardless of sex, were used for isolation of P0, P4 and P7 islets. Both the adult and postnatal mice used were wild-type mice on a C57BL/6J background (C57BL/6JRj, Janvier Labs, France). We used 12–13-week-old male *db/db* mice for isolation of *db/db* islets; age-matched male black lean control *db/+* mice were used for *db/+* islets. Both *db/db* and *db/+* mice were on a BKS background [BKS(D)-*Lepr*^*db*^/JOrlRj, Janvier Labs].

For the in vivo treatment, 8-week-old male *db/db* mice on a BKS background [BKS(D)-*Lepr*^*db*^/JOrlRj, Janvier Labs] were used. Adjudin (50 mg/kg per mouse, HY-18996, MedChemExpress, USA), which was dissolved in DMSO (D2650, Sigma, USA) and then mixed with corn oil (HY-Y1888, MedChemExpress) at a dilution of 1:9, or vehicle (DMSO mixed with corn oil at a dilution of 1:9) was injected intraperitoneally into 8-week-old male *db/db* mice every other day for 3 weeks. Body weight and blood glucose levels were monitored weekly. For *db/db* mice with body weight over 40 g at 8 weeks old, 100 mg/kg per mouse of adjudin was used for treatment.

### Chemical treatments, beta cell ablation and glucose assay of zebrafish larvae

Adjudin (HY-18996, MedChemExpress) was added to E3 medium to give a final concentration of 5 µmol/l for treating zebrafish larvae. We ablated the beta cells in *Tg(ins:Flag-NTR)* or *Tg(ins:CFP-NTR)* zebrafish larvae by incubating the larvae in E3 medium supplemented with 10 mmol/l metronidazole (MTZ) (M3761, Sigma), 1% DMSO (23500.297, VWR, USA) and 0.2 mmol/l 1-phenyl-2-thiourea (PTU) (P7629, Sigma) from 3 to 4 days post-fertilisation (dpf). Free glucose levels were determined by lysing each larva and analysing the lysate using the Glucose Colorimetric/Fluorometric Assay Kit (K688, BioVision, USA). The genotype of *ins* mutant zebrafish larvae was determined by real-time PCR with the previously published *ins* primers 5′-GTGCTCTGTTGGTCCTGTTGG-3′ and 5′-CATCGACCAGATGAGATCCACAC-3′ [22]. The genotyping analysis was done by comparing the shape of the melting curve of the samples with that of the wild-type zebrafish genomic DNA.

### Immunostaining of zebrafish larvae

The number of beta cells was examined in *Tg(ins:H2BGFP)* zebrafish. Immunostaining was performed according to standard procedures and analysed with a Leica SP8 confocal microscope (Germany). The whole endocrine portion of the pancreas was scanned in all larvae analysed. Confocal stacks were analysed with Fiji software (https://fiji.sc, version: 1.54f). The anti-GFP antibody was used to enhance the fluorescence of GFP (1:500, GFP-1020, Aves Labs, USA).

### Luciferase assay for zebrafish larvae

Luciferase assay was used to examine *ins* promoter activity in *Tg(ins:NLuc)* zebrafish. For zebrafish larvae during beta cell regeneration, three larvae per well were plated in 96-well plates with 200 µl of E3 medium and treated with DMSO or 5 µmol/l adjudin from 4 to 5 dpf, after ablation of beta cells from 3 to 4 dpf. Zebrafish larvae were anaesthetised with tricaine (Sigma) at 5 dpf, and 50 μl of Nano-Glo Luciferase reagent (N1110, Promega, USA) was added to each well. The luminescence signals were recorded using a Glomax-96 microplate luminometer (GM3000, Promega, USA) after 1.5 h of incubation at room temperature. The *z* score was calculated as (luminescence value [treated well]−mean luminescence value [DMSO-treated wells]) × (SD [DMSO-treated wells])^−1^. For zebrafish in the basal state, one larva per well was plated in 96-well plates with 200 µl of E3 medium and treated with DMSO or 5 µmol/l adjudin from 4 to 5 dpf, followed by anaesthetisation and analysis of luminescence signals.

### Live calcium imaging of zebrafish beta cells

Imaging of pancreatic beta cells was performed with 5 dpf *Tg(ins:GCaMP6s);Tg(ins:H2BmCherry);Tg(ins:Flag-NTR)* zebrafish larvae using a ZEISS LSM 900 confocal microscope equipped with a W Plan-Apochromat ×20/1 NA (numerical aperture) water correction lens (ZEISS, Germany). Signal from one focal plane per islet was recorded. After beta cell ablation from 3 to 4 dpf, the zebrafish larvae were treated with DMSO or 5 µmol/l adjudin from 4 to 5 dpf before imaging. The GCaMP6s and mCherry signals from beta cells were simultaneously acquired using the 488-nm and 587-nm laser lines. The GCaMP6s signal was rendered in green and the mCherry signal was rendered in red. Time series recordings were taken with an in-plane resolution of 1024×1024 pixels. The videos were recorded for 200 cycles, with approximately 2 s acquisition time per frame. Videos were analysed in ImageJ (https://imagej.net/ij/download.html, version: 1.53t).

### Quantitative real-time PCR

Total RNA was extracted using Trizol (15596018, Thermo Fisher Scientific, USA) or Quick-RNA Microprep Kit (R1051, Zymo Research, USA). cDNA for quantitative real-time PCR (qPCR) experiments was synthesised with High Capacity cDNA Reverse Transcription Kit (4368813, Applied Biosystems, USA), and relative mRNA expression levels were determined by using iTaq Universal SYBR Green Supermix (1725124, Bio-Rad, USA) and a ViiA 7 Real-Time PCR System (Applied Biosystems). For zebrafish, mRNA levels were normalised to the *eef1a1l1* mRNA levels. Sequences of primers used for zebrafish are listed in electronic supplementary material (ESM) Table 1. For mouse islets, *Actb* or *Tbp* was used as the housekeeping gene to normalise gene expression. Sequences of primers used for mice are listed in ESM Table 2.

### Islet isolation and treatment

To isolate islets from adult mice, collagenase P (COLLP-RO, Sigma) was added to Hanks’ balanced salt solution (14025050, Thermo Fisher Scientific) at a concentration of 0.2 mg/ml and injected into the common bile duct to inflate the whole pancreas. The inflated pancreas was dissected out and digested in 0.2 mg/ml collagenase P for 8 min in a water bath at 37°C. After several washes, the islets were handpicked and left in an incubator for overnight recovery. Islets from P0, P4 and P7 mice were isolated based on a previously published protocol [23]. In short, the pancreas was dissected out and cut into small pieces, followed by digestion, washes, handpicking and overnight recovery. After that, islets were transferred to the medium with DMSO or 10 µmol/l adjudin for the treatment. For proliferation assessments, 20 µmol/l EdU (E10415, Thermo Fisher Scientific) was added to islets for 2 h incubations at the end of the treatment. The medium used for islet culture was RPMI 1640 medium (21875034, Thermo Fisher Scientific) supplemented with 11 mmol/l glucose, 10% FBS (26140079, Thermo Fisher Scientific) and 1% penicillin-streptomycin (10378016, Thermo Fisher Scientific).

For P0, P4 and P7 islets, pancreases from 6–9 male or female mice were used for islet isolation and considered as one biological sample for either DMSO or adjudin treatment. For islets from wild-type adult, *db/db* and *db/+* mice, one biological sample corresponded to islets from 1–2 male mice. We used islets from 12–13-week-old *db/db* mice such that they would contain dedifferentiated beta cells, and if a batch of islets were aberrant they were not used.

### GSIS in islet cultures

After treatment with compounds, islets were washed twice with pre-warmed Krebs–Ringer bicarbonate HEPES buffer (KRBH) (J67795, Thermo Fisher Scientific, containing 2.8 mmol/l glucose, 120 mmol/l sodium chloride, 5 mmol/l potassium chloride, 2 mmol/l calcium chloride, 1 mmol/l magnesium chloride, 25 mmol/l sodium bicarbonate, 5.5 mmol/l HEPES and 0.1% BSA) and incubated in KRBH at 37°C for 1 h. Islets from each group were subsequently transferred to KRBH containing 2.8 mmol/l glucose for 1 h, then to 16.7 mmol/l glucose for another 1 h, and the supernatant from each incubation was collected for ELISA (90080, Crystal Chem, USA) analysis. The islets were lysed with RIPA lysis buffer (R0278, Sigma) containing protease inhibitor (11697498001, Roche, Switzerland) and phosphatase inhibitor (4906845001, Roche) cocktails. Islet insulin content was quantified using ELISA (90080, Crystal Chem). Protein levels were then measured with the Pierce BCA Protein Assay kit (23227, Thermo Fisher Scientific) to normalise secreted insulin and islet insulin content.

### Whole mount staining for mouse islets

Mouse islets were washed with cold PBS and fixed in 4% formaldehyde on ice for 30 min. After two washes in PB (2.5% BSA in PBS), islets were left in blocking solution containing 5% normal donkey serum, 1% BSA, 0.5% Triton X-100 and 5% DMSO in PBS for 1 h at room temperature or overnight at 4°C and then incubated with primary antibody for 48–72 h at 4°C. The primary antibodies used were anti-insulin (no dilution, IR00261, Agilent, USA), anti-PDX1 (1:100, ab47267, Abcam, UK), anti-NKX6.1 (1:100, F55A10, DSHB, USA), anti-MafA (1:100, NBP1-00121, Novusbio, USA), anti-insulin (1:500, 16049, ProGen, Germany), anti-TOM20 (translocase of outer mitochondrial membrane 20) (1:100, ab56783, Abcam) and anti-E-cadherin (1:500, 3195, Cell Signaling Technology [CST], USA). The islets were then washed three times in PB, incubated with secondary antibodies at 4°C for 24–48 h and mounted in RapiClear 1.47 (RC147001, SunJin Lab, Taiwan) with a circular well iSpacer (IS204, SunJin Lab) for microscopy [24]. The whole islets were scanned with a Leica SP8 confocal microscope and confocal stacks were analysed with Fiji software. The Click-iT EdU Alexa Fluor 647 imaging kit (C10340, Thermo Fisher Scientific) was used to detect proliferative cells.

### Western blot for mouse islets

Islets were collected using RIPA buffer (R0278, Sigma) supplemented with protease (11697498001, Roche) and phosphatase inhibitors (4906845001, Roche). Samples were mixed with Laemmli buffer (1610737, Bio-Rad) and run on precast 4–15% gradient gels (4561086, Bio-Rad) on a Mini-PROTEAN 3 (Bio-Rad) at 100 V (constant) and then transferred to nitrocellulose membranes for 30 min using the Trans-Blot Turbo Transfer system (Bio-Rad). Next, nitrocellulose membranes were blocked with 5% skim milk (70166, Sigma) for 1 h at room temperature and incubated for 48–72 h at 4°C with the following primary antibodies: anti-TOM20 (1:500, 11802-1-AP, Proteintech, USA), anti-E-cadherin (1:500, 3195, CST), anti-TFAM (transcription factor A, mitochondrial) (1:1000, ab131607, Abcam) and anti-β-actin (1:500, A5441, Sigma). Secondary antibodies (115-036-146 and 711-036-152, Jackson ImmunoResearch, UK) were added at a 1:5000 dilution and incubated at room temperature for 1 h. Blots were developed using SuperSignal West Dura Extended Duration Substrate (37071, Thermo Fisher Scientific) and imaged with the ChemiDoc Imaging system (Bio-Rad). Quantification of western blots was performed in Fiji.

### Glucose uptake in zebrafish larvae

For zebrafish larvae in the basal state, the larvae were treated with 5% glucose (G8270, Sigma) from 4 dpf to 5 dpf. After that, glucose was washed out, the larvae were treated with DMSO or 5 µmol/l adjudin from 5 to 6 dpf and 20 µmol/l 2-NBDG [2-(*N*-(7-nitrobenz-2-oxa-1,3-diazol-4-yl)amino)-2-deoxyglucose] (N13195, Thermo Fisher Scientific) was added to the medium for overnight incubation at the end of treatment. For zebrafish larvae during beta cell regeneration, the *Tg(ins:Flag-NTR)* larvae were incubated with MTZ to ablate beta cells from 3 to 4 dpf, then treated with DMSO or 5 µmol/l adjudin from 4 to 5 dpf, and 20 µmol/l 2-NBDG was added to the medium for around 8 h of incubation at the end of treatment. After being euthanised by tricaine (Sigma), the larvae were immobilised in 2% methylcellulose (M0387, Sigma). 2-NBDG fluorescence signals were analysed with a Leica SP8 confocal microscope. The *ins* mutant was analysed in the same way, but after the microscopy the genotype of *ins* mutant zebrafish larvae was determined by real-time PCR as described in the section ‘Chemical treatments, beta cell ablation and glucose assay of zebrafish larvae’.

### RNA-seq and data analysis

Total RNA was extracted using the Quick-RNA Microprep Kit (R1051, Zymo Research) and sent to Novogene (UK) for cDNA library construction and high-throughput sequencing. Paired-end sequencing was carried out with the Illumina NovaSeq (paired-end 150 nucleotide read length). Clean reads were aligned to the mouse genome (GRCm38/mm10) using HISAT2 (v2.0.5, https://daehwankimlab.github.io/hisat2/). The abundance of each transcript was quantified using featureCounts (v1.5.0-p3, https://subread.sourceforge.net/featureCounts.html). Differential expression analysis was performed using the DESeq2 R package (1.20.0, https://bioconductor.org/packages/release/bioc/html/DESeq2.html). The Gene Ontology (GO) enrichment analyses were performed with the R package clusterProfiler (https://bioconductor.org/packages/release/bioc/html/clusterProfiler.html). The principal components were calculated based on the top 5000 variable genes determined by median absolute deviation. The principal component analysis (PCA) plots were built using ggord (https://github.com/fawda123/ggord/) and rgl (https://github.com/dmurdoch/rgl) R packages. Heatmaps were generated using the pheatmap R package (https://github.com/raivokolde/pheatmap). The local version of the gene set enrichment analysis (GSEA) analysis tool (http://www.broad.mit.edu/gsea) was used to identify the enriched molecular signatures archived in the Molecular Signatures Database (MsigDB) (http://www.gsea-msigdb.org/gsea/msigdb) [25, 26]. All the analyses were performed in R version 3.2.5 (http://www.r-project.org).

### Immunohistochemistry for mouse pancreas

Pancreases were fixed in 4% formaldehyde overnight at 4°C, embedded in paraffin, then sectioned. Slides were deparaffinised using xylene and rehydrated using a descending series of alcohol to distilled water. Slides were permeabilised in 0.3% Triton X-100 in PBS for 10 min followed by rinsing with distilled water. Antigen retrieval was performed in a microwave oven by heating sections in target retrieval solution (S236984, Dako, Denmark) for 8 min. Sections were washed twice with PBST (0.1% Triton X-100 in PBS) after they had cooled to room temperature, blocked in antibody diluent (S302283-2, Agilent) for 1 h at room temperature and incubated with primary antibodies overnight at 4°C. The primary antibodies used were anti-insulin (1:5, IR00261, Agilent), anti-PDX1 (1:400, ab47267, Abcam) and anti-NKX6.1 (1:100, F55A10, DSHB). The sections were then washed with PBST, incubated with secondary antibodies at room temperature for 2 h and mounted for microscopy.

To calculate beta cell mass, immunohistochemistry for insulin was performed on six evenly spaced sections throughout the entire pancreas, tiled images were taken from each section with an ImageXpress Pico microscope (Molecular Devices, USA), fluorescent area of insulin was determined as a percentage of the total pancreatic area (CellRepoterXpress, Molecular Devices) and beta cell mass was calculated with fluorescent area of insulin multiplied by the weight of the pancreas. An image of each islet was taken with a Zeiss LSM980-Airy, and intensity and percentage of NKX6.1 and PDX1 in beta cells were analysed in Fiji in a blinded manner; 13 to 23 islets per mouse were examined.

### Intraperitoneal glucose tolerance test and intraperitoneal glucose stimulated insulin secretion

The intraperitoneal glucose tolerance test (IPGTT) was performed as described previously [27]. Briefly, mice were fasted overnight and injected with 0.5 g/kg glucose intraperitoneally. Blood glucose levels were measured from the tail vein at 0 min, 7.5 min, 15 min, 30 min, 45 min, 60 min, 90 min and 120 min after the injection. Blood samples were collected at 0 min, 15 min, 30 min, 60 min and 120 min to examine plasma insulin levels with ELISA (90080, Crystal Chem).

### Primary human hepatocyte culture

Cryopreserved primary human hepatocytes (PHHs) were obtained with prior consent from one male donor through a commercial supplier (BioIVT, US). 3D PHHs were cultured as described previously [28]. Briefly, cells were thawed and seeded in ultra-low-attachment plates (Corning, USA) with a density of 1500 viable cells per well. William’s E medium (A1217601, Thermo Fisher Scientific, USA) was used for seeding and maintenance, and was supplemented with 11 mmol/l glucose, 100 nmol/l dexamethasone, 5.5 mg/l transferrin, 6.7 μg/l selenite, 2 mmol/l l-glutamine, 1 U/ml penicillin, 0.1 mg/ml streptomycin, 10% FBS with 1.7 μmol/l insulin. The cells naturally aggregated to form spheroids within 7 days, and then FBS was phased out. The spheroids were conditioned for 3 weeks before being exposed to 0 (DMSO), 3 or 10 μmol/l adjudin for 24 h. Medium was replenished every 2–3 days.

For experiments in ESM Fig. 8b,c, PHHs were cultured in the same medium but with 0.1 nmol/l insulin. Cells aggregated within 7 days, and then insulin and FBS were phased out prior to treatment with 0 (DMSO), 3, 10 or 30 μmol/l adjudin for 24 h.

### Glucose consumption measurement in PHHs

Culture medium was collected from each treatment group. Cell-free medium was collected at the same time point to measure the background glucose concentration in the medium. The glucose uptake was quantified with a sensitive colorimetric method as previously described [29].

### Statistical analysis

The samples were randomised. Zebrafish were randomly picked for DMSO or adjudin treatment. Mouse islets were randomly picked for treatment. Islets were size matched between treatments. In vivo experiments were performed in male *db/db* mice, which were randomly treated either with vehicle or adjudin.

**Fig. 1:**
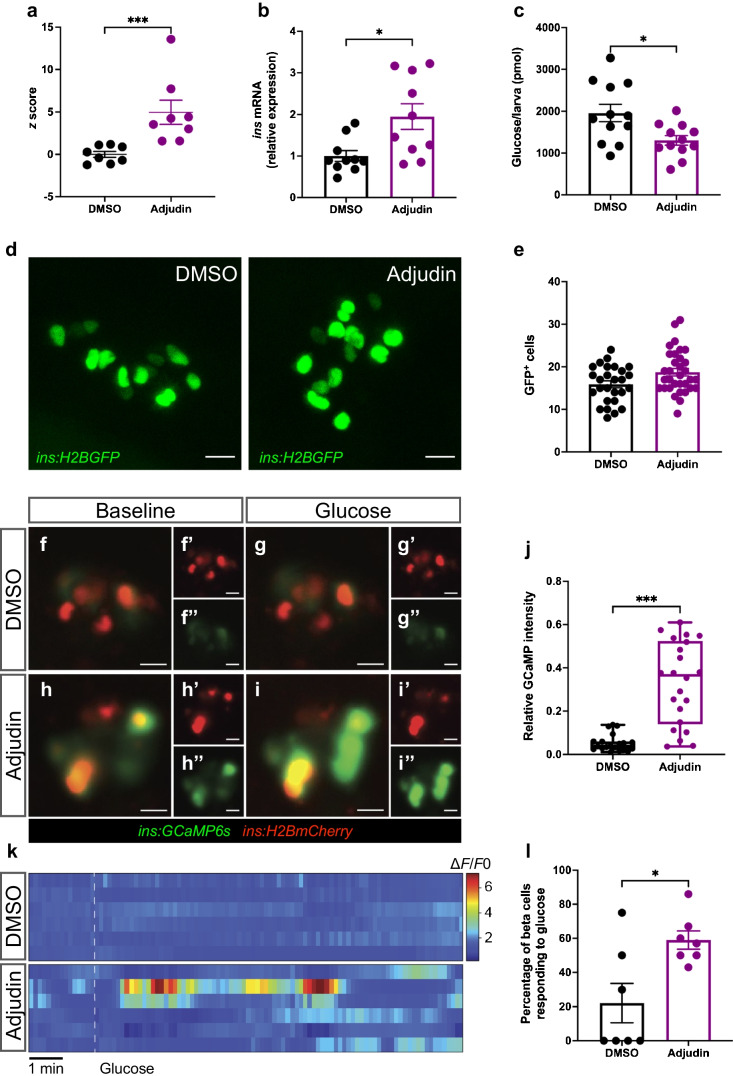
Adjudin improves the function of regenerated beta cells in zebrafish. (**a**) The *z* score of bioluminescence during beta cell regeneration. *Tg(ins:NLuc);Tg(ins:Flag-NTR)* larvae were treated with DMSO or 5 µmol/l adjudin for 1 day after beta cell ablation, and bioluminescence was analysed at 5 dpf. Mann–Whitney test: ***p<0.001. *n*=24 larvae per treatment (3 larvae per data point). Data are presented as mean ± SEM. (**b**) qPCR analysis of *ins* expression in zebrafish larvae during beta cell regeneration. After beta cell ablation, *Tg(ins:Flag-NTR)* larvae were treated with DMSO or 5 µmol/l adjudin for 1 day from 4 to 5 dpf before being analysed by qPCR. Mann–Whitney test: *p<0.05. *n*=80 larvae per treatment (5–12 larvae per data point). Data are presented as mean ± SEM. (**c**) Free glucose levels in zebrafish larvae during beta cell regeneration. *Tg(ins:CFP-NTR)* larvae were treated with DMSO or 5 µmol/l adjudin for 2 days after beta cell ablation, and free glucose levels were analysed at 6 dpf. Mann–Whitney test: *p<0.05. *n*=12 larvae per treatment. Data are presented as mean ± SEM. (**d**) Maximum projections of primary islets in zebrafish during beta cell regeneration. *Tg(ins:H2BGFP);Tg(ins:Flag-NTR)* larvae were treated with DMSO or 5 µmol/l adjudin for 2 days after beta cell ablation, and subsequently fixed at 6 dpf for analysis. Scale bar, 10 μm. (**e**) Quantification of total number of regenerated beta cells in (**d**). Mann–Whitney test. *n*=27 larvae (DMSO), *n*=33 larvae (adjudin). Data are presented as mean ± SEM. (**f**–**i**) Representative images from live calcium recording of islets before (**f** and **h**) and after the addition of 200 mmol/l glucose to the E3 medium (**g** and **i**). Beta cells expressing H2BmCherry are shown in red and the calcium signal in green. Images with merged channels are shown in (f, g, h, i), single red channels are shown in (f’, g’, h’, i’), and single green channels are shown in (f’’, g’’, h’’, I’’). *Tg(ins:GCaMP6s);Tg(ins:H2BmCherry);Tg(ins:Flag-NTR)* zebrafish larvae were treated with DMSO or 5 µmol/l adjudin for 1 day after beta cell ablation from 3 to 4 dpf, and imaged at 5 dpf. Scale bar, 10 µm. (**j**) Quantification of relative GCaMP intensity in beta cells at baseline (**f** and **h**). Mann–Whitney test: ***p<0.001. *n*=20 cells from four larvae (DMSO), *n*=22 cells from four larvae (adjudin). (**k**) Calcium activity indicated by normalised fluorescence over time in regenerated beta cells. Each line represents one cell. The addition of 200 mmol/l glucose is indicated by the white dashed line. *Tg(ins:GCaMP6s);Tg(ins:H2BmCherry);Tg(ins:Flag-NTR)* zebrafish larvae were treated with DMSO or 5 µmol/l adjudin for 1 day after beta cell ablation from 3 to 4 dpf, and had calcium signal recorded at 5 dpf. *n*=6 cells per treatment. (**l**) Quantification of percentage of beta cells that had calcium response to glucose. *Tg(ins:GCaMP6s);Tg(ins:H2BmCherry);Tg(ins:Flag-NTR)* zebrafish larvae were treated with DMSO or 5 µmol/l adjudin for 1 day after beta cell ablation from 3 to 4 dpf, and imaged at 5 dpf. Mann–Whitney test: *p<0.05. *n*=7 larvae per treatment. Data are presented as mean ± SEM. Δ*F*/*F*0, signal-to-baseline ratio; Δ*F*, deviation from the baseline;* F*0, basal fluorescence

Results are presented as mean values ± SEM. GraphPad Prism (version 9.4.1) was used for statistical analysis. Figure 1k and ESM Fig. 1 were made using Python with the library Matplotlib [30]. The statistical tests used are listed in the corresponding figure legends; *p* values < 0.05 are considered significant.

## Results

### Adjudin improves the function of regenerated beta cells in zebrafish

In an effort to find novel ways to recover functional beta cell mass in diabetes, based on a transgenic zebrafish model, *Tg(ins:Flag-NTR)*, in which nitroreductase (NTR) is expressed under *ins* promoter and turns chemical MTZ into toxic products to induce apoptosis of beta cells, we previously performed in vivo chemical screens and identified hits that promoted beta cell proliferation [31, 32] and differentiation [33, 34]. Here, combined with another transgenic zebrafish model in which the *ins* promoter drives the expression of nanoluciferase, *Tg*(*ins:Nluc*), we found that one hit from the screening, adjudin, increased the bioluminescent signal (Fig. 1a) and *ins* mRNA expression (Fig. 1b) and decreased glucose levels in zebrafish larvae (Fig. 1c) during beta cell regeneration, without affecting the number of regenerated beta cells (Fig. 1d,e). Since calcium influx triggers insulin secretion, live imaging was performed in *Tg(ins:GCaMP6s);Tg(ins:H2BmCherry);Tg(ins:Flag-NTR)* zebrafish larvae to monitor beta cell calcium activity (Fig. 1f–i). At 1 day after ablation, we found that regenerated beta cells had significantly higher GCaMP intensity at baseline with adjudin treatment (Fig. 1j). Upon glucose challenge, the percentage of beta cells having calcium response was 22% in fish with DMSO treatment, which was increased to 59% by adjudin (Fig. 1k,l, ESM Videos 1, 2). Because adjudin has been reported as a potential blocker of chloride channels [17], and to see whether it has an acute effect on beta cells, different concentrations of adjudin were applied to challenge 5 dpf zebrafish larvae. Calcium signals increased and showed that around 40% of beta cells had a calcium response within 5 min upon 10 µmol/l adjudin challenge, and 85–95% of the beta cells had an even stronger response upon 50 µmol/l challenge (ESM Fig. 1, ESM Video 3).

The results suggest that, instead of increasing the number of regenerated beta cells, adjudin increased the function of regenerated beta cells in vivo in zebrafish larvae.

### Adjudin improves the function of neonatal mouse islets

**Fig. 2:**
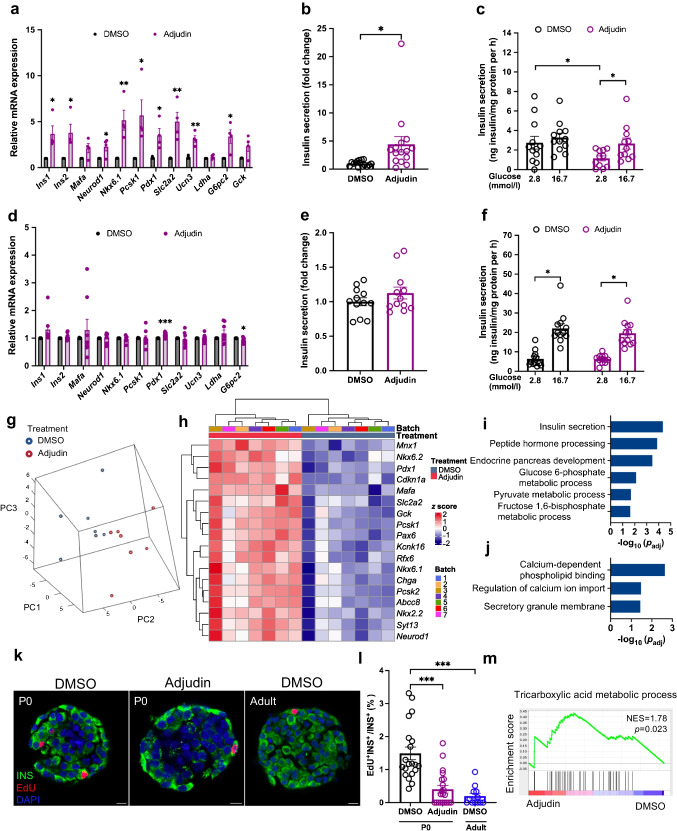
Adjudin improves the function of neonatal mouse islets. (**a**) qPCR analysis of the expression of beta cell maturation markers in P0 islets. P0 islets were cultured in medium containing 11 mmol/l glucose and treated with DMSO or 10 µmol/l adjudin for 1 day before qPCR. Student’s *t* test: *p<0.05, **p<0.01 vs DMSO. *n*=4 per treatment. Data are presented as mean ± SEM. (**b**) Accumulated insulin secretion by P0 islets. P0 islets were cultured in medium containing 11 mmol/l glucose and treated with DMSO or 10 µmol/l adjudin for 1 day. Culture medium was collected at the end of the treatment to analyse insulin levels. Student’s *t* test: *p<0.05. *n*=15 per treatment. Data are presented as mean ± SEM. (**c**) GSIS of P0 islets. P0 islets were cultured in medium containing 11 mmol/l glucose and treated with DMSO or 10 µmol/l adjudin for 1 day before GSIS. Student’s *t* test: *p<0.05. *n*=12 (DMSO), *n*=11 (adjudin). Data are presented as mean ± SEM. (**d**) qPCR analyses of the expression of beta cell maturation markers in adult islets. Adult islets were cultured in medium containing 11 mmol/l glucose and treated with DMSO or 10 µmol/l adjudin for 1 day before qPCR. Student’s *t* test: *p<0.05, ***p<0.001 vs DMSO. *n*=8 per treatment. Data are presented as mean ± SEM. (**e**) Accumulated insulin secretion by adult islets. Adult islets were cultured in medium containing 11 mmol/l glucose and treated with DMSO or 10 µmol/l adjudin for 1 day. Culture medium was collected at the end of the treatment to analyse insulin levels. Student’s *t* test. *n*=12 per treatment. Data are presented as mean ± SEM. (**f**) GSIS of adult islets. Adult islets were cultured in medium containing 11 mmol/l glucose and treated with DMSO or 10 µmol/l adjudin for 1 day before GSIS. Student’s *t* test: *p<0.05. *n*=12 per treatment. Data are presented as mean ± SEM. (**g**) 3D PCA plot for P0 islets. (**h**) Heatmap of significantly upregulated genes related to beta cell function in P0 islets after adjudin treatment, the scale represents *z* score. (**i**, **j**) GO terms associated with upregulated genes after adjudin treatment in P0 islets. (**i**) GO terms related to beta cell development and function and glucose metabolism. (**j**) GO terms related to downstream signalling of GSIS. (**k**) Representative immunofluorescence images from whole mount staining of mouse islets to assess proliferation. Islets were cultured in medium containing 11 mmol/l glucose and treated with DMSO or 10 µmol/l adjudin for 1 day. A final concentration of 20 µmol/l EdU was used for 2 h of incubation at the end of the treatment. Scale bar, 10 µm. (**l**) Quantification of percentage of proliferative beta cells in (**k**). One-way ANOVA with Tukey’s multiple comparisons test: ***p<0.001. *n*=20 (P0, DMSO), *n*=21 (P0, adjudin), *n*=12 (adult, DMSO). Data are presented as mean ± SEM. (**m**) GSEA plot showing gene sets related to ‘tricarboxylic acid metabolic process’. INS, insulin; NES, normalised enrichment score; PC1, principal component 1; PC2, principal component 2; PC3, principal component 3

To translate our findings to higher species and to test whether islets are a direct target of adjudin, we examined the effects of adjudin on pancreatic islets isolated from both P0 and adult (3-month-old) mice (hereafter called P0 islets and adult islets). All islets were cultured in medium containing 11 mmol/l glucose and treated with either DMSO or adjudin for 1 day to examine the mRNA expression of markers of functional beta cells and the actual function of the islets. In P0 islets, many genes were upregulated after adjudin treatment, including *Ins1*, *Ins2*, beta cell transcription factors (*Neurod1*, *Nkx6.1* and *Pdx1*), the proinsulin processing gene *Pcsk1*, the glucose transporter *Slc2a2*, the glucose metabolic gene *G6pc2* and the beta cell maturation gene *Ucn3* (Fig. 2a). Culture medium was collected at the end of the treatment to compare accumulated insulin secretion between the two groups, and a fourfold increase in insulin secretion was found in the culture medium of P0 islets after treatment with adjudin (Fig. 2b), indicating that those islets become more functional during adjudin treatment. To further assess functional changes in islets, GSIS was performed. P0 islets did not respond to glucose stimulation in the DMSO group as expected (Fig. 2c), while adjudin treatment led to glucose responsive insulin secretion mainly by decreasing basal insulin secretion (Fig. 2c), a feature of adult islets (ESM Fig. 2a). No significant difference was found in insulin content in P0 islets between the two groups (ESM Fig. 2b). In adult islets, a slight increase in *Pdx1* expression and a slight decrease in *G6pc2* expression were found after adjudin treatment (Fig. 2d), but no significant difference was observed in accumulated insulin secretion (Fig. 2e), GSIS (Fig. 2f) or islet insulin content (ESM Fig. 2c), suggesting no functional effect of adjudin on adult islets.

Next, RNA-seq was performed in P0 islets to compare global transcriptional changes between islets treated with DMSO and adjudin. Seven biological replicates from each group were sequenced and analysed. Islets were segregated by treatment in a 3D PCA plot (Fig. 2g). The expression of 1395 genes was significantly changed (|log_2_fold change|>0, *p*<0.05), of which 853 genes were upregulated by adjudin, and 542 were downregulated (ESM Fig. 2d, ESM Tables 3, 4). Among the markers of functional beta cells, beta cell transcription factors (e.g. *Mnx1*, *Nkx6.1*, *Nkx6.2*, *Pdx1*, *Mafa*, *Pax6*, *Rfx6*, *Nkx2.2* and *Neurod1*), glucose metabolic genes (e.g. *Slc2a2* and *Gck*), proinsulin processing genes (*Pcsk1* and *Pcsk2*), ion channel genes (e.g. *Kcnk16* and *Abcc8*) and genes involved in insulin vesicle formation and exocytosis (e.g. *Chga* and *Syt13*) were significantly upregulated after adjudin treatment (Fig. 2h, ESM Fig. 2e). Beta cell disallowed genes like *Hk1*, *Hk2*, *Slc16a1*, *Ldha*, *Rest*, *Pdgfra*, *Oat* and *Cxcl12* were not significantly changed, except that *Igfbp4* was upregulated (ESM Fig. 2f). Consistent with the functional changes we found earlier, the GO analysis (ESM Tables 5, 6) showed that the upregulated genes were significantly enriched in biological processes including insulin secretion, peptide hormone processing and endocrine pancreas development (Fig. 2i). Enrichment of upregulated genes in biological processes such as the glucose 6-phosphate metabolic process, pyruvate metabolic process and fructose 1,6-bisphosphate metabolic process (Fig. 2i) indicates that adjudin treatment led to increased glucose metabolism in P0 islets. Adjudin-upregulated genes were also associated with biological processes involved in downstream signalling of GSIS, indicated by enrichment of GO terms such as ‘regulation of calcium ion import’, ‘calcium-dependent phospholipid binding’ and ‘secretory granule membrane’ (Fig. 2j).

To validate the changes of functional markers at the protein level, whole mount staining was performed to examine expression of PDX1, NKX6.1 and MafA in P0 islets (ESM Fig. 2g). The percentage of beta cells expressing PDX1 in adult islets (99.67%) was slightly higher compared with P0 islets (98.45%), although no significant difference was found in P0 islets treated with DMSO or adjudin (ESM Fig. 2h). The percentage of beta cells expressing NKX6.1 and MafA in P0 islets with DMSO treatment was 98.06% (ESM Fig. 2i) and 82.99% (ESM Fig. 2j), respectively, and no significant changes were observed for either marker when compared with adjudin-treated P0 or DMSO-treated adult islets. The results indicate that the percentage of beta cells expressing those transcription factors in P0 islets is comparable to adult islets, and does not change with adjudin treatment.

Functional maturation is coupled with beta cells becoming less proliferative [35]. In our RNA-seq data, the cell cycle activator *Pdgfrb* was significantly downregulated by adjudin (ESM Fig. 3a), cell cycle inhibitors *Cdkn1a* and *Cdkn1c* were upregulated and *Cdkn2a* was downregulated (Fig. 2h, ESM Fig. 3b). To assess proliferation of beta cells, EdU incorporation experiments were performed. As expected, beta cells in adult islets were much less proliferative compared with P0 islets (Fig. 2k,l). P0 islets had 1.49% proliferating beta cells, which adjudin treatment significantly decreased to 0.41% (Fig. 2k,l). As proliferation of mouse beta cells peaks at P4–P7 [36], we tested whether adjudin had an effect on those islets, by first evaluating mRNA expression of beta cell functional markers. Adjudin treatment resulted in increased expression of *Neurod1*, *Pcsk1* and *Gck*, and slightly decreased *Nkx6.1* and *Ucn3* (ESM Fig. 3c). The extent of increased gene expression was less compared with that in P0 islets. EdU incorporation showed that P4 and P7 islets had around 5% proliferating beta cells, and that adjudin treatment decreased beta cell proliferation in P4 islets, but not in P7 islets (ESM Fig. 3d–f). Thus, these results suggest that the maturation effects of adjudin may decrease as mice age and are not dependent on a high proliferation rate, but rather that the maturation leads to a decrease in proliferation.

Mitochondrial metabolism is important for beta cell function. In GSEA (ESM Tables 7, 8), we found that gene sets related to the TCA metabolic process (Fig. 2m, ESM Fig. 4a), 2-oxoglutarate metabolic process (ESM Fig. 4b,c) and NADH metabolic process (ESM Fig. 4d,e) are upregulated by adjudin, suggesting increased mitochondrial function in P0 islets. Next, we examined the expression of genes involved in oxidative phosphorylation (Complex I–V) (ESM Fig. 4f–j) and genes encoding inner (ESM Fig. 4k) and outer mitochondrial membrane transport proteins (ESM Fig. 4l) and mitochondrial ribosomal proteins (ESM Fig. 4m), and no significant changes were observed. Among the mitochondrial genes, expression levels of transfer RNA *mt-Tc*, *mt-Te*, mitochondrially encoded 16S RNA *mt-Rnr2* and mitochondrially encoded NADH dehydrogenase 1 *mt-Nd1* were significantly upregulated after adjudin treatment (ESM Fig. 4n). Expression levels of mitochondrial outer membrane protein TOM20 and mitochondrial transcription factor A TFAM were examined in islets. Even though adult islets had higher expression levels of TOM20 (ESM Fig. 5a–c) and TFAM (ESM Fig. 5b,d), no significant difference was seen in P0 islets between the two treatments.

Because adjudin has been reported to disrupt specific cell–cell junctions in the testis [16], we also explored this in islets. In our RNA-seq data, genes involved in cell–cell contact like *Cdh1* and *Cldn4* were upregulated, and *Itga5* was downregulated (ESM Fig. 5f). To test whether it was affected by adjudin, E-cadherin was examined: adult islets had increased expression of E-cadherin, but no difference was observed in P0 islets (ESM Fig 5b,e,g). Moreover, because adjudin is a potent blocker of chloride channels [17], we mined this dataset and found that the gene encoding the voltage-gated calcium-activated chloride channel, *Ano1*, was upregulated by adjudin, and the Solute Carrier Family 4 Member 1 gene *Slc4a1* was downregulated (ESM Fig. 5h). However, these correlations are unlikely to be causative with regard to the effect of adjudin.

Overall, these data indicate that adjudin treatment enhanced the function of P0 islets in vitro, but decreased beta cell proliferation in P0 islets. In contrast, no functional changes were found in adult islets with adjudin treatment.

### Adjudin improves the recovery of islets from db/db mice

**Fig. 3:**
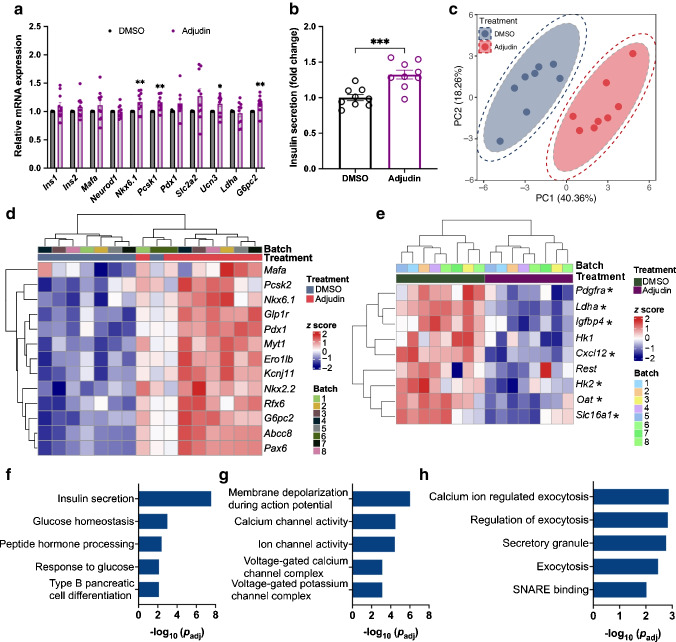
Adjudin improves the function of islets from *db/db* mice. (**a**) qPCR analysis of the expression of beta cell maturation markers in *db/db* islets. The *db/db* islets were cultured in medium containing 11 mmol/l glucose and treated with DMSO or 10 µmol/l adjudin for 1 day before qPCR. Student’s *t* test: *p<0.05, **p<0.01 vs DMSO. *n*=10 per treatment. Data are presented as mean ± SEM. (**b**) Accumulated insulin secretion by *db/db* islets. The *db/db* islets were cultured in medium containing 11 mmol/l glucose and treated with DMSO or 10 µmol/l adjudin for 1 day. Culture medium was collected at the end of the treatment to analyse insulin levels. Student’s *t* test: ***p<0.001. *n*=9 per treatment. Data are presented as mean ± SEM. (**c**) PCA plot for *db/db* islets. The inside and outside dashed lines indicate 95% and 98% CI. (**d**) Heatmap of significantly upregulated genes related to beta cell function in *db/db* islets after adjudin treatment, the scale represents *z* score. (**e**) Heatmap of beta cell disallowed genes in *db/db* islets after adjudin treatment, the scale represents *z* score. *Significantly regulated genes,* p*<0.05. (**f**–**h**) GO terms associated with upregulated genes after adjudin treatment in *db/db* islets. (**f**) GO terms related to beta cell development and function. (**g**) GO terms related to membrane ion channels. (**h**) GO terms related to exocytosis. PC1, principal component 1; PC2, principal component 2

Islets from type 2 diabetes have been shown to be dysfunctional and dedifferentiated [6–8]. To test whether adjudin can recover the function of islets from diabetic mice, we used *db/db* mice, a severe type 2 diabetic model that carries a spontaneous mutation in the leptin receptor. As islets from *db/db* mice (hereafter called *db/db* islets) were shown to be dedifferentiated at 12–13 weeks [37–39], islets at the same stage were isolated and treated with DMSO or adjudin. Islets at this stage are dedifferentiated, as expression levels of markers of functional beta cells, like *Ins1*, *Ins2*, *Mafa*, *Neurod1*, *Nkx6.1*, *Pcsk1*, *Pdx1*, *Slc2a2*, *G6pc2* and *Ucn3*, were substantially decreased compared with islets of *db/+* mice, and expression levels of beta cell disallowed genes like *Ldha* and *Hk1* increased in *db/db* islets (ESM Fig. 6a). Adjudin treatment led to a significant increase in the expression of *Nkx6.1*, *Pcsk1*, *G6pc2* and *Ucn3* in *db/db* islets (Fig. 3a). By the end of the treatment, a significant increase of accumulated insulin secretion was observed in the culture medium of adjudin-treated *db/db* islets (Fig. 3b). In GSIS experiments, neither basal insulin secretion, nor insulin secretion upon glucose challenge (ESM Fig. 6b), nor insulin content (ESM Fig. 6c) was significantly changed by adjudin in *db/db* islets.

To better understand the global transcriptional profile, RNA-seq was conducted on *db/db* islets. Eight biological samples from each group were sequenced and analysed: 2873 genes were changed significantly by adjudin (|log_2_fold change|>0, *p*<0.05), among which 1430 were upregulated and 1443 were downregulated (ESM Fig. 6d, ESM Tables 9, 10). Islets were segregated by treatment in a PCA plot (Fig. 3c). We found that the beta cell transcription factors (e.g. *Nkx6.1*, *Pdx1*, *Myt1*, *Mafa*, *Pax6*, *Rfx6* and *Nkx2.2*), glucose metabolic genes (e.g. *G6pc2*), proinsulin processing genes (e.g. *Pcsk2* and *Ero1lb*), ion channel genes (e.g. *Kcnj11* and *Abcc8*), genes involved in exocytosis (e.g. *Syt7* and *Syt13*), beta cell maturation marker *Ucn3* and glucagon-like peptide-1 receptor *Glp1r* were among the significantly upregulated genes after adjudin treatment (Fig. 3d, ESM Fig. 6e). Beta cell disallowed genes like *Hk2*, *Slc16a1*, *Ldha*, *Pdgfra*, *Igfbp4*, *Cxcl12* and *Oat* were significantly downregulated by adjudin (Fig. 3e). GO analysis (ESM Tables 11, 12) showed that upregulated genes were significantly enriched in biological processes including insulin secretion, glucose homeostasis, peptide hormone processing, response to glucose and type B pancreatic cell differentiation (Fig. 3f). Moreover, adjudin-upregulated genes were associated with biological processes and cellular components involved in membrane ion channels, such as membrane depolarisation during action potential, calcium channel activity, ion channel activity, voltage-gated calcium channel complex and voltage-gated potassium channel complex (Fig. 3g). Biological processes related to exocytosis were also among the GO terms of upregulated genes, such as calcium ion-regulated exocytosis, regulation of exocytosis, secretory granule, exocytosis and SNARE (soluble *N*-ethylmaleimide-sensitive factor attachment protein receptor) binding (Fig. 3h). In GSEA (ESM Tables 13, 14), gene sets related to type B pancreatic cell differentiation (ESM Fig. 6f,g) and positive regulation of insulin secretion involved in cellular response to glucose stimulus (ESM Fig. 6h,i) were upregulated by adjudin.

Next, we examined genes associated with various important cellular events. With regard to proliferation, we found that the expression levels of cell cycle activator genes *Cdk1*, *Ccna2*, *Pdgfra* and *Ccnb2* were decreased, and *Ccnd1* and *Ccnd2* were increased by adjudin (ESM Fig. 7a), whereas there were no significant changes in cell cycle inhibitor genes (ESM Fig. 7b). Some genes involved in oxidative phosphorylation were decreased, including *Ndufa2*, *Ndufa4*, *Ndufa5*, *Ndufa6*, *Ndufa13*, *Ndufs7* and *Ndufs8* in Complex I; *Uqcrb* in Complex III; *Cox5a*, *Cox5b*, *Cox6b1* and *Cox7b* in Complex IV; and *Atp5b*, *Atp5c1* and *Atp5l* in Complex V (ESM Fig. 7c–g). Among genes encoding inner mitochondrial membrane transport proteins, *Slc25a29* was upregulated, whereas *Slc25a4* was downregulated by adjudin (ESM Fig. 7h). *Tomm22* in the outer mitochondrial membrane was decreased (ESM Fig. 7i). Genes encoding mitochondrial ribosomal proteins, *Mrpl12* and *Mrpl39*, were decreased (ESM Fig. 7j). Among the mitochondrial genes, *mt-Co1* and *mt-Co2* (encoding subunits of Complex IV), *mt-Rnr1* and *mt-Rnr2* (mitochondrially encoded ribosomal RNA), *mt-Nd1* and *mt-Nd2* (encoding subunits of NADH dehydrogenase), *mt-Ty*, *mt-Tc* and *mt-Tn* (transfer RNA) were upregulated, and *mt-Tp*, *mt-Cytb* (encoding subunits of oxidative phosphorylation Complex III), *mt-Nd4*, *mt-Nd5* and *mt-Nd6* were downregulated (ESM Fig. 7k). Cell–cell contact genes including *Cdh2*, *Robo1* and *Robo2* were upregulated, whereas *Cldn4* and *Itgb1* were downregulated (ESM Fig. 7l). Genes associated with chloride channels including *Slc12a2*, *Slc12a5*, *Slc12a7*, *Cftr* and *Lrrc8d* were upregulated by adjudin (ESM Fig. 7m). Although these changes in expression may indicate the state of the islet, they need to be further validated for functional importance. Together, these results suggest that the recovery of *db/db* islets was improved by adjudin treatment.

### Adjudin stimulates glucose uptake in the zebrafish liver and in human liver spheroids

**Fig. 4:**
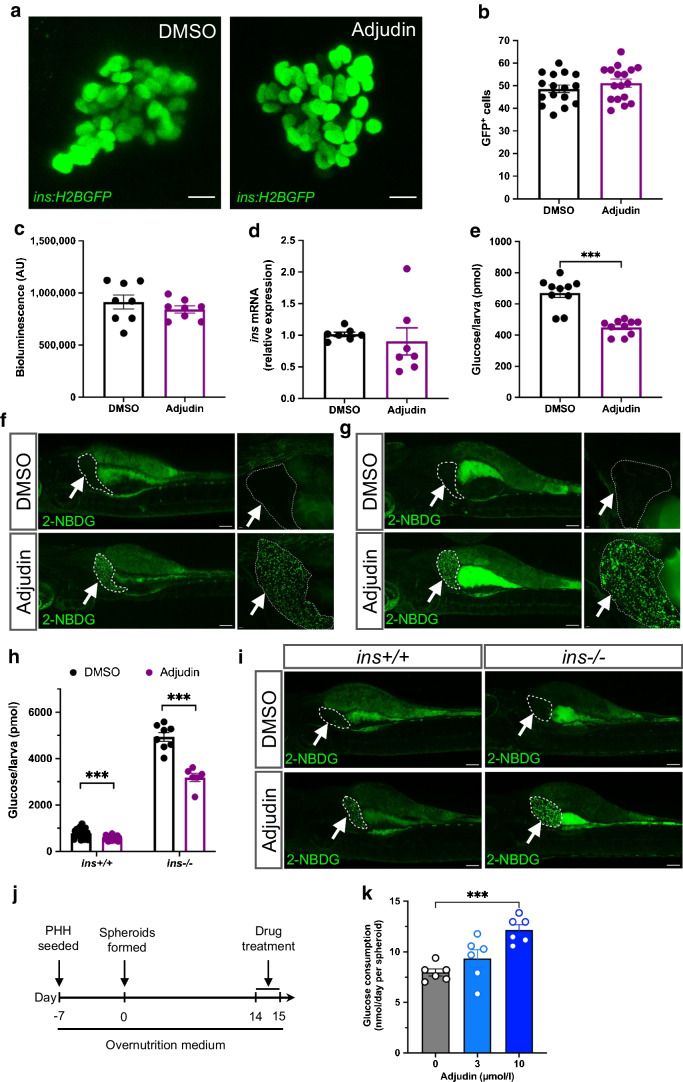
Adjudin stimulates glucose uptake in the zebrafish liver and in human liver spheroids. (**a**) Maximum projections of primary islets in zebrafish larvae in the basal state (i.e. in the absence of beta cell ablation). *Tg(ins:H2BGFP)* larvae were treated with DMSO or 5 µmol/l adjudin for 2 days and fixed at 6 dpf for analysis. Scale bar, 10 μm. (**b**) Quantification of total number of beta cells in zebrafish larvae in the basal state (**a**). Mann–Whitney test. *n*=16 larvae (DMSO), *n*=17 larvae (adjudin). Data are presented as mean ± SEM. (**c**) Bioluminescence of zebrafish larvae in the basal state. *Tg(ins:NLuc)* larvae were treated with DMSO or 5 µmol/l adjudin for 1 day, and the luciferase assay was performed at 5 dpf. Mann–Whitney test. *n*=8 larvae per treatment. Data are presented as mean ± SEM. (**d**) qPCR analysis of *ins* expression in zebrafish larvae in the basal state. Wild-type zebrafish larvae were treated with DMSO or 5 µmol/l adjudin for 1 day before being analysed by qPCR at 5 dpf. Mann–Whitney test. *n*=65 larvae per treatment (5–12 larvae per data point). Data are presented as mean ± SEM. (**e**) Free glucose levels in zebrafish larvae in the basal state. Zebrafish larvae were treated with DMSO or 5 µmol/l adjudin for 2 days, and the free glucose levels were analysed at 6 dpf. Mann–Whitney test. ***p<0.001. *n*=10 larvae per treatment. Data are presented as mean ± SEM. (**f**) Glucose uptake indicated by 2-NBDG. Zebrafish larvae were pretreated with 5% glucose for 1 day, followed by treatment with DMSO or 5 µmol/l adjudin for 1 day, then 20 µmol/l 2-NBDG was added at the end of the treatment and the fluorescence analysed at 6 dpf. Livers of the larvae are outlined by white dashed lines and pointed to by white arrows. Scale bar, 100 µm (left panel); scale bar, 10 µm (right panel). (**g**) Glucose uptake indicated by 2-NBDG in zebrafish during beta cell regeneration. *Tg(ins:Flag-NTR)* zebrafish larvae were treated with DMSO or 5 µmol/l adjudin for 1 day following beta cell ablation, then 20 µmol/l 2-NBDG was added at the end of the treatment and the fluorescence signals were analysed at 5 dpf. Livers of the larvae are outlined by white dashed lines and pointed to by white arrows. Scale bar, 100 µm (left panel); scale bar, 10 µm (right panel). (**h**) Free glucose levels in *ins +/+* and *ins −/−* larvae. *ins +/−* zebrafish were incrossed to generate *ins +/+* and *ins −/−* larvae. The larvae were treated with DMSO or 5 µmol/l adjudin for 2 days from 3 to 5 dpf prior to analysis of free glucose levels. Mann–Whitney test: ***p<0.001. *n*=19 larvae (*ins +/+*, DMSO), *n*=19 larvae (*ins +/+*, adjudin), *n*=8 larvae (*ins −/−*, DMSO), *n*=6 larvae (*ins −/−*, adjudin). Data are presented as mean ± SEM. (**i**) Glucose uptake indicated by 2-NBDG in *ins+/+* (left panel) and *ins−/−* (right panel) zebrafish. The zebrafish larvae were treated with DMSO or 5 µmol/l adjudin for 1 day, then 20 µmol/l 2-NBDG was added at the end of the treatment and the fluorescence signals were analysed at 5 dpf. Livers of the larvae are outlined by white dashed lines and pointed to by white arrows. Scale bar, 100 µm. (**j**) Schematic showing the timeline of experiments using PHHs. The PHHs were seeded to form spheroids and conditioned in overnutrition medium for 3 weeks to induce insulin resistance prior to treatment with DMSO, 3 µmol/l adjudin or 10 µmol/l adjudin. The glucose level in the medium was measured before and after the treatment. (**k**) Glucose consumption of the PHH spheroids treated as described in (**j**). One-way ANOVA followed by Dunnett’s multiple comparisons test: ***p<0.001. *n*=6 independent biological replicates per treatment. Data are presented as mean ± SEM. AU, arbitrary units

In the basal state of zebrafish larvae (in which beta cells were not ablated), adjudin did not change the number of beta cells (Fig. 4a,b). Moreover, in this setting neither *ins* promoter activity (Fig. 4c) nor insulin mRNA expression (Fig. 4d) was significantly changed by adjudin. However, a reduction in glucose levels was observed after adjudin treatment (Fig. 4e), suggesting adjudin can target other tissues beside beta cells in vivo. To investigate peripheral effects of adjudin and to examine how glucose was reduced, 2-NBDG, a fluorescent glucose analogue, was added at the end of the treatment to monitor glucose distribution in zebrafish larvae pretreated with 5% glucose. Surprisingly, 2-NBDG signals were clearly found in livers of adjudin-treated larvae (Fig. 4f), suggesting the liver may play a role in glucose clearance in the basal state. At this point, we wondered whether adjudin had any effects on the liver of zebrafish in which beta cells were ablated. To test this hypothesis, zebrafish larvae were treated with adjudin after beta cell ablation, and 2-NBDG was added at the end of the treatment. Similarly, 2-NBDG signals showed a clear increase of glucose uptake in livers of adjudin-treated larvae (Fig. 4g).

To examine the dependence on insulin, adjudin was tested in a previously described insulin-deficient mutant that carries a 16 bp deletion in *ins*, resulting in homozygous mutant zebrafish lacking insulin protein in pancreatic islets [22]. Compared with the *ins +/+* control siblings, *ins −/−* mutant larvae exhibited as expected a dramatic increase in the glucose level (ESM Fig. 8a, Fig. 4h). Adjudin treatment significantly reduced glucose levels in both control siblings and *ins* mutants (Fig. 4h), suggesting that the suppression of glucose by adjudin can be insulin independent. Next, 2-NBDG was applied to those zebrafish larvae, showing that adjudin promoted glucose uptake in liver even in *ins −/−* mutants (Fig. 4i). To assess the direct effect of adjudin on liver cells, we employed a 3D PHH in vitro model that exhibits physiologically relevant responses to insulin challenge and nutritional perturbation [29]. First, PHH spheroids were conditioned in low physiological insulin levels and high glucose levels for a week, followed by insulin phasing-out (ESM Fig. 8b). A tendency of increased glucose consumption was found in PHH spheroids after adjudin treatment (ESM Fig. 8c).

Next, to investigate effects of adjudin on human hepatocytes similar to those in type 2 diabetes, PHH spheroids were conditioned in overnutrition medium for 3 weeks to induce insulin resistance (Fig. 4j) [29], followed with treatment of either 0 (DMSO), 3 or 10 µmol/l adjudin. Glucose consumption was significantly and dose-dependently increased by about 50% (from 8±0.8 to 12.2±1.3 nmol/day per spheroid) compared with untreated PHH spheroids (Fig. 4k). Together, these results indicate that adjudin promoted glucose consumption in PHH spheroids with insulin resistance, and can exert a conserved glucose-lowering effect across species.

### Adjudin improves glucose homeostasis in db/db mice

**Fig. 5:**
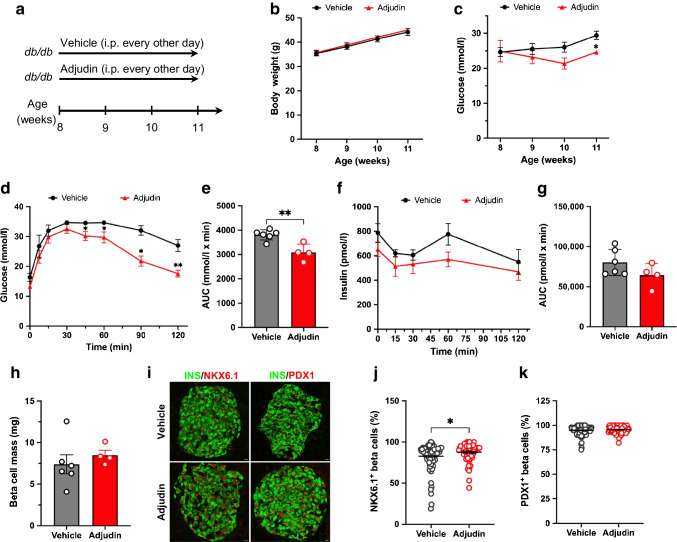
Adjudin improves glucose homeostasis in *db/db* mice. (**a**) Schematic of the in vivo study using 8-week-old male *db/db* mice. The mice received intraperitoneal injections of vehicle (*n*=6) or 50 mg/kg adjudin (*n*=4) every other day for 3 weeks. Body weight and nonfasting glucose were measured weekly. IPGTT and histological examination were performed at the end of the treatment. (**b**) Weekly body weight of the *db/db* mice. Student’s *t* test. Data are presented as mean ± SEM. (**c**) Weekly nonfasting blood glucose of the *db/db* mice. Student’s *t* test: *p<0.05 vs vehicle. Data are presented as mean ± SEM. (**d**) IPGTT. Student’s *t* test: *p<0.05, **p<0.01 vs vehicle. Data are presented as mean ± SEM. (**e**) AUC of the IPGTT. Student’s *t* test: **p<0.01. Data are presented as mean ± SEM. (**f**) Intraperitoneal glucose stimulated insulin secretion (IPGSIS). Blood insulin levels in the IPGTT experiment. Student’s *t* test. Data are presented as mean ± SEM. (**g**) AUC curve of the IPGSIS. Student’s *t* test. Data are presented as mean ± SEM. (**h**) Pancreatic beta cell mass in vehicle- and adjudin-treated *db/db* mice. Student’s *t* test. Data are presented as mean ± SEM. (**i**) Immunofluorescence staining of insulin (INS), NKX6.1 and PDX1 in pancreas from vehicle- and adjudin-treated *db/db* mice. Scale bar, 10 µm. (**j**) Quantification of the percentage of insulin-positive cells expressing NKX6.1 per islet. Student’s *t* test. *p<0.05. *n*=101 islets (vehicle), *n*=67 islets (adjudin). Data are presented as mean ± SEM. (**k**) Quantification of the percentage of insulin-positive cells expressing PDX1 per islet. Student’s *t* test. *n*=90 islets (vehicle), *n*=65 islets (adjudin). Data are presented as mean ± SEM

Based on the in vitro results, we decided to test the possible therapeutic effect of adjudin in diabetic mice. *db/db* mice on the BKS background become glucose intolerant by 8 weeks of age (phenotype information for BKS-db, The Jackson Laboratory, USA). Therefore, 8-week-old *db/db* mice were injected intraperitoneally with either vehicle or adjudin every other day for 3 weeks. Body weight and nonfasting blood glucose were monitored weekly, and IPGTT was performed by the end of the treatment (Fig. 5a). No difference in body weight was seen between the two groups during the treatment (Fig. 5b). Nonfasting blood glucose was gradually decreasing in *db/db* mice during the first 2 weeks of treatment with adjudin, and was significantly downregulated after 3 weeks of treatment (Fig. 5c). In IPGTT, the blood glucose level in mice treated with adjudin was significantly lower starting from 45 min after the glucose challenge until the end of the experiment, compared with mice injected with vehicle (Fig. 5d,e). Blood samples were collected during IPGTT to examine insulin levels, and there was no significant difference between the two groups (Fig. 5f,g). To determine whether adjudin affects beta cells in *db/db* mice, pancreases collected from those mice were analysed. No significant difference in beta cell mass was found between the two groups (Fig. 5h). The intensity of NKX6.1 (Fig. 5i, ESM Fig. 9a) and PDX1 (Fig. 5i, ESM Fig. 9b) was slightly increased after adjudin treatment, as well as the percentage of beta cells expressing NKX6.1 (Fig. 5j). No significant difference was found in the percentage of beta cells expressing PDX1 (Fig. 5k). These results suggest that adjudin treatment improved glucose homeostasis in *db/db* mice.

## Discussion

Adjudin is a multifunctional drug that is a potential reversible male contraceptive [16] and a chloride channel blocker [17] and has anti-cancerogenic [18], anti-neuroinflammation [19] and anti-oxidative stress [20] effects. In this study, we expand the known biological activities of adjudin to include glucose-lowering effects by targeting both the pancreatic islets and the liver.

Functional maturation of beta cells is the process in which beta cells gain the capability to respond to glucose. Diet switch from fat-rich milk to carbohydrate-rich chow during weaning in mice has been shown to be coupled with changes in expression of microRNAs [40], and a switch of signalling pathways from the nutrient sensor target of rapamycin (mTORC1) to 5′-adenosine monophosphate-activated protein kinase (AMPK) [41]. This results in changes of metabolic enzymes, increases in mitochondria and oxidative phosphorylation, which eventually leads to functional maturation of beta cells [42]. DNA methyltransferase 3 alpha (DNMT3A)-mediated inhibition of the disallowed genes including *Hk1* and *Ldha* [43], and oestrogen-related receptor γ (ERRγ)-mediated increases in oxidative phosphorylation, electron transport chain and ATP production [44], were shown to be involved in the metabolic switch from predominantly glycolysis to oxidative phosphorylation in the maturation of beta cells. Functional maturation of beta cells also involves biogenesis and maturation of insulin vesicles as regulated by calcineurin/NFAT (nuclear factor of activated T cells) signalling [45], and changes in calcium sensitivity of insulin vesicles as regulated by synaptotagmins [46]. Several efforts have been made to identify compounds that can promote beta cell maturity and function. BRD7552 was found as a *PDX1* inducer in human ductal carcinoma cell line PANC-1 and primary human islets [47]. Carbamazepine, inhibitor of the Nav1.7 sodium channel, was identified in MIN6 beta cells and validated in primary mouse islets as a stimulator of *Ins1* and *Ins2* expression [48]. Based on expression of the beta cell maturation marker urocortin 3 (UCN3) [3, 49], screening on mouse islets found that the activin receptor-like kinase 5 (ALK5) inhibitor II can prevent islets from losing expression of transcription factors under stressed conditions [50]. Screening using stem cell-derived beta-like cells revealed that the rho-associated protein kinase (ROCK) inhibitor H1152 promoted maturation of human beta-like cells [51]. Screening for modulators of *Pdx1* expression in zebrafish identified the histone deacetylase (HDAC) inhibitor HC toxin, which also promoted the expression of *Pdx1* and *Ins1* in MIN6 cells, and of *PDX1*, *NKX2.2*, *NEUROD1* and *INS* in stem cell-derived beta-like cells, and enhanced the function of primary mouse and human islets [52]. Here, we showed that the small molecule adjudin, which was identified in an in vivo zebrafish beta cell screen, improved the function of newly regenerated beta cells in vivo in zebrafish. This effect was conserved in mice, as adjudin also increased markers of functional beta cells and increased accumulated insulin secretion in P0 mouse islets. The process of functional maturation is accompanied by decreased proliferation and, consistently, we found adjudin decreased beta cell proliferation in P0 islets. Moreover, these islets gained the capability of glucose responsive insulin secretion after treatment with adjudin. Considering that stem cell-derived islets (SC-islets) are functionally immature [53, 54], adjudin might be useful to improve functionality of SC-islets for cell therapy. As beta cells are dedifferentiated in type 2 diabetes [6–8], and preserving beta cell function by therapeutics has been shown to be beneficial to glucose homeostasis in diabetes [55], we extended our studies to in vitro isolated islets from *db/db* mice. Adjudin treatment of *db/db* islets resulted in increased expression of functional beta cell markers, decreased expression of beta cell disallowed genes and increased accumulated insulin secretion. However, adjudin had no effect on GSIS, which may depend on protein levels of the functional markers and the secretory machinery. RNA-seq of *db/db* islets showed that adjudin increased biological processes associated with membrane ion channel activity and exocytosis.

Our study found that the liver was another target of adjudin. Adjudin treatment boosted glucose uptake in liver of zebrafish larvae both in the basal state and following beta cell ablation. Incorporation of 2-NBDG into glycogen makes it florescent [56]; thus, the signals from liver of zebrafish might indicate increased glycogen synthesis during adjudin treatment. Changes of glucose uptake in muscles were not observed in zebrafish larvae. In agreement with the findings from zebrafish, we found that adjudin increased glucose consumption in PHH spheroids with insulin resistance.

Moreover, we tested adjudin in a previously described *ins* mutant [22]. Adjudin treatment significantly reduced glucose levels in both control siblings and *ins* mutants, suggesting that the suppression of glucose by adjudin is at least partially insulin independent. 2-NBDG signals showed that adjudin promoted glucose uptake in liver even in *ins* mutant larvae. Findings from insulin-sensitive PHH spheroids in the presence of high glucose showed that adjudin increased glucose consumption even after phasing-out of insulin. These results indicate that adjudin has insulin-independent effects on glucose uptake, in both zebrafish and human liver cells. Previous studies of in vitro cultured human hepatocytes have shown that 55% of hepatic glucose uptake is mediated by insulin, 45% of which is insulin independent [29], and our results suggest that adjudin can promote glucose uptake in an insulin-independent manner.

The effects of adjudin on pancreatic islets and liver led us to test it in *db/db* mice. Nonfasting blood glucose was decreased after 3 weeks of treatment with adjudin (24.6 mmol/l) compared with that in vehicle-treated mice (29.4 mmol/l), although it was not restored to the level of similarly aged *db/+* mice (8.9 mmol/l based on Jaxpheno17 from The Jackson Laboratory). The mice treated with adjudin cleared glucose faster with relatively less insulin, indicating a decreased dependency on insulin. Moreover, even though blood insulin levels were not significantly changed during IPGTT by adjudin, an increased percentage of beta cells expressed NKX6.1, which could be a direct effect of adjudin. Alternatively, this effect could be indirect as glucose disposal by other tissues decreases stress on beta cells, which is beneficial for functional recovery of the beta cells [57]. Muscles and adipose tissues were not examined in our experiments, and thus the possibility of them being additional targets of adjudin cannot be excluded and needs to be further investigated.

In conclusion, we identified the small molecule adjudin in an in vivo chemical screening using zebrafish, and showed that adjudin enhanced the function of immature or dysfunctional islets and stimulated glucose uptake in liver. Moreover, adjudin improved glucose homeostasis in *db/db* mice. Thus, adjudin may serve as a proof-of-concept compound that targets several different tissues to improve glucose homeostasis in diabetes.

### Supplementary Information

Below is the link to the electronic supplementary material.

ESM (PDF 1301 KB)

ESM Tables (XLSX 705 KB)

**ESM Video 1.** **Live calcium imaging of beta cells in a DMSO- treated larva after 1 day of beta cell regeneration. Related to Fig. 1f and 1h.**
An example video of live calcium imaging of beta cells in a *Tg(ins:GCaMP6s);Tg(ins:H2BmCherry);Tg(ins:Flag-NTR)* zebrafish larva at 5 dpf after 1 day of beta cell regeneration, with beta cells expressing H2BmCherry shown in red, calcium signal in green. The zebrafish larvae were treated with DMSO for 1 day after beta cell ablation from 3-4 dpf, and had calcium signal recorded at 5 dpf. (MP4 11998 KB) 

**ESM Video 2.** **Live calcium imaging of beta cells in a Adjudin-treated larva after 1 day of beta cell regeneration. Related to Fig. 1g and 1i.**
An example video of live calcium imaging of beta cells in a *Tg(ins:GCaMP6s);Tg(ins:H2BmCherry);Tg(ins:Flag-NTR)* zebrafish larva at 5 dpf after 1 day of beta cell regeneration, with beta cells expressing H2BmCherry shown in red, calcium signal in green. The zebrafish larvae were treated with Adjudin for 1 day after beta cell ablation from 3-4 dpf, and had calcium signal recorded at 5 dpf. (MP4 13709 KB)

**ESM Video 3.** **Live calcium imaging of beta cells in a larva subjected to acute treatment with Adjudin. Related to ESM Fig.1.**
An example video of live calcium imaging of beta cells in a *Tg(ins:GCaMP6s);Tg(ins:H2BmCherry)* fish at 5 dpf, with beta cells expressing H2BmCherry shown in red, calcium signal in green. The zebrafish larvae were treated with 5% glucose from 3-4 dpf, then glucose was washed out, and calcium signal was recorded at 5 dpf before and after acute treatment with Adjudin. (MP4 13533 KB)

## Data Availability

Raw and processed RNA-seq data for this study have been deposited in the Gene Expression Omnibus under accession number GSE235398 (https://www.ncbi.nlm.nih.gov/geo/query/acc.cgi?acc=GSE235398).

## Abbreviations

Dpf: Days post-fertilisation
GO: Gene Ontology
GSEA: Gene set enrichment analysis
GSIS: Glucose stimulated insulin secretion
IPGTT: Intraperitoneal glucose tolerance test
KRBH: Krebs–Ringer bicarbonate HEPES buffer
MafA: MAF bZIP transcription factor A
MTZ: Metronidazole
2-NBDG: 2-(*N*-(7-Nitrobenz-2-oxa-1,3-diazol-4-yl)amino)-2-deoxyglucose
NKX6.1: NK6 homeobox 1
NTR: Nitroreductase
P: Postnatal day
PB: 2.5% BSA in PBS
PBST: 0.1% Triton X-100 in PBS
PCA: Principal component analysis
PDX1: Pancreatic and duodenal homeobox 1
PHH: Primary human hepatocyte
qPCR: Quantitative real-time PCR
SC-islet: Stem cell-derived islet
SNARE: Soluble *N*-ethylmaleimide-sensitive factor attachment protein receptor
TCA: Tricarboxylic acid
TFAM: Transcription factor A, mitochondrial
TOM20: Translocase of outer mitochondrial membrane 20
