# Supplementary material for: Adjudin improves beta cell maturation, hepatic glucose uptake and glucose homeostasis
Source: Diabetologia. 2023 Oct 16;67(1):137–55. doi: 10.1007/s00125-023-06020-4 (PMC10709271; doi:10.1007/s00125-023-06020-4)
Supplement: Supplementary file 1 — ESM (PDF 1301 KB) [file 125_2023_6020_MOESM1_ESM.pdf]

Electronic Supplementary Material

Adjudin improves beta cell maturation, hepatic glucose uptake and glucose homeostasis

Lipeng Ren, Jérémie Charbord, Lianhe Chu, Aurino M Kemas, Maria Bertuzzi, Jiarui Mi, Chen Xing, Volker M Lauschke, Olov Andersson

Contents:

ESM Table 1 & 2
ESM Figure 1-9
ESM Video 1-3 legends

ESM Table 1. Primers used for qPCR of zebrafish genes

	fwd	rev
eef1a1l1	GTGCTGTGCTGATTGTTGCT	TGTATGCGCTGACTTCCTTG
ins	TCTGGTCGATGCCCTTTATC	ATCAGCTCGGCATGATCTTT

ESM Table 2. Primers used for qPCR of mouse genes

	fwd	rev
Actb	GGCTGTATTCCCCTCCATCG	CCAGTTGGTAACAATGCCATGT
Tbp	CTGGAATTGTACCGCAGCTT	ATGATGACTGCAGCAAATCG
Ins1	CAGAGACCATCAGCAAGCAG	GGGACCACAAAGATGCTGTT
Ins2	GTCAAGCAGCACCTTTGTGGTTCC	ACAATGCCACGCTTCTGCTG
Mafa	CTCCAGAGCCAGGTGGAG	GTACAGGTCCCGCTCCTTG
Neurod1	GCCCAGCTTAATGCCATCTTT	CAAAAGGGCTGCCTTCTGTAA
Nkx6.1	AAAACACACCAGACCCACGTT	TTCTGGAACCAGACCTTGACC
Pdx1	TCCACCACCACCTTCCAGCTCA	AATTCCTTCTCCAGCTCCAG
Slc2a2	GGGCCATCAACATGATCTTC	AATCATCCCGGTTAGGAACA
Pcsk1	TGGAGTTGCATATAATTCCAAAGTT	CTAGCCTCAATGGCATCAGTT
Ucn3	GCTGTGCCCCTCGACCT	TGGGCATCAGCATCGCT
Ldha	CCGTTACCTGATGGGAGAGA	GTAGGCACTGTCCACCACCT
G6pc2	CCCTGATGGTGGTGGCTCTA	GTCTGTGGGTGGAGCAGGAC
Hk1	CGGAATGGGGAGCCTTTGG	GCCTTCCTTATCCGTTTCAATGG

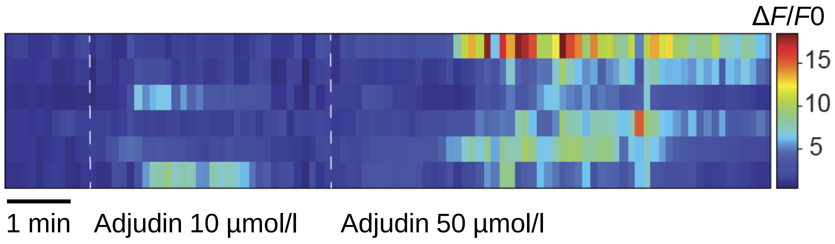

ESM Figure 1

ESM Fig. 1 Acute effects of Adjudin on beta cells in zebrafish. Related to Fig.1.

Calcium activity indicated by normalized fluorescence over time in beta cells. Each line represents one cell. *Tg(ins:GCaMP6s);Tg(ins:H2BmCherry)* zebrafish larvae were treated with 5% glucose from 3 to 4 dpf, then glucose was washed out and live calcium imaging was performed at 5 dpf. The addition of 10 µmol/l and 50 µmol/l Adjudin is indicated by the white dashed lines. Each line represents one cell. n=6 cells from 1 larva.

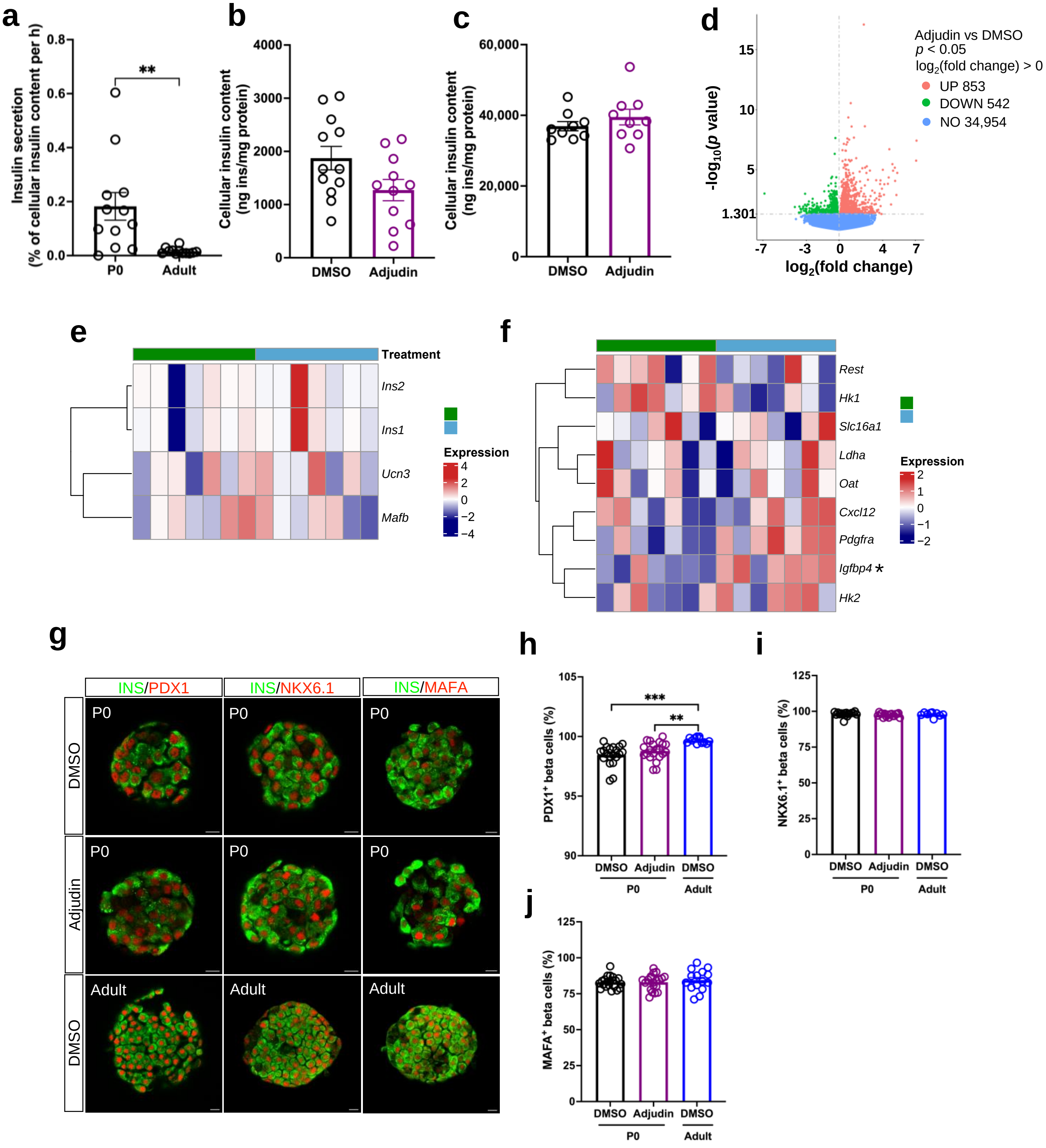

a
Insulin secretion (% of cellular insulin content per h)
0.0
0.2
0.4
0.6
0.8
**
P0
Adult
b
Cellular insulin content (ng ins/mg protein)
0
1000
2000
3000
4000
DMSO
Adjudin
c
Cellular insulin content (ng ins/mg protein)
0
20,000
40,000
60,000
DMSO
Adjudin
d
Adjudin vs DMSO
p < 0.05
log2(fold change) > 0
UP 853
DOWN 542
NO 34,954
-log10(p value)
1.301
5
10
15
-7
-3
0
4
7
log2(fold change)
e
Treatment
Ins2
Ins1
Ucn3
Mafb
Expression
4
2
0
−2
−4
f
Rest
Hk1
Slc16a1
Ldha
Oat
Cxcl12
Pdgfra
Igfbp4 *
Hk2
Expression
2
1
0
−1
−2
g
INS/PDX1
INS/NKX6.1
INS/MAFA
DMSO
Adjudin
DMSO
P0
Adult
h
PDX1+ beta cells (%)
90
95
100
105
***
**
DMSO
Adjudin
DMSO
P0
Adult
i
NKX6.1+ beta cells (%)
0
25
50
75
100
125
DMSO
Adjudin
DMSO
P0
Adult
j
MAFA+ beta cells (%)
0
25
50
75
100
125
DMSO
Adjudin
DMSO
P0
Adult

ESM Figure 2

ESM Fig. 2 Effects of Adjudin on P0 and adult islets. Related to Fig. 2.

(a) Basal insulin secretion from GSIS shown in percentage of cellular insulin content. Student's t test. n=12 per treatment. $^{**}p < 0.01$. Data are presented as mean ± SEM.

(b) Insulin content in P0 islets. P0 islets were cultured in medium containing 11 mmol/l glucose and treated with DMSO or 10 μmol/l Adjudin for 1 day before GSIS, and islet insulin content was measured at the end of the GSIS. Student's t test. n=12 (DMSO), n=11 (Adjudin). Data are presented as mean ± SEM.

(c) Insulin content in adult islets. Adult islets were cultured in medium containing 11 mmol/l glucose and treated with DMSO or 10 μmol/l Adjudin for 1 day before GSIS, and islet insulin content was measured at the end of the GSIS. Student's t test. n=12 per treatment. Data are presented as mean ± SEM.

(d) Volcano plot for P0 islets.

(e-f) Heatmap of beta cell maturation markers (e) and disallowed genes (f) in P0 islets. * indicates significantly regulated genes.

(g) Representative immunofluorescence images of whole mount staining of mouse islets to assess the expression of PDX1, NKX6.1 and MAFA in beta cells. Islets were cultured in medium containing 11 mmol/l glucose and treated with DMSO or 10 μmol/l Adjudin for 1 day before fixation and whole mount staining. Scale bar=10 μm.

(h-j) Quantification of percentage of beta cells expressing PDX1 (h), NKX6.1 (i), MAFA (j) in (g). One-way ANOVA with Tukey's multiple-comparisons test. $^{**}p < 0.01$, $^{***}p < 0.001$. For PDX1 (h), n=20 (P0, DMSO), n=21 (P0, Adjudin), n=12 (adult, DMSO). For NKX6.1 (i), n=17 (P0, DMSO), n=17 (P0, Adjudin), n=9 (adult, DMSO). For MAFA (j), n=21 (P0, DMSO), n=20 (P0, Adjudin), n=15 (adult, DMSO). Data are presented as mean ± SEM.

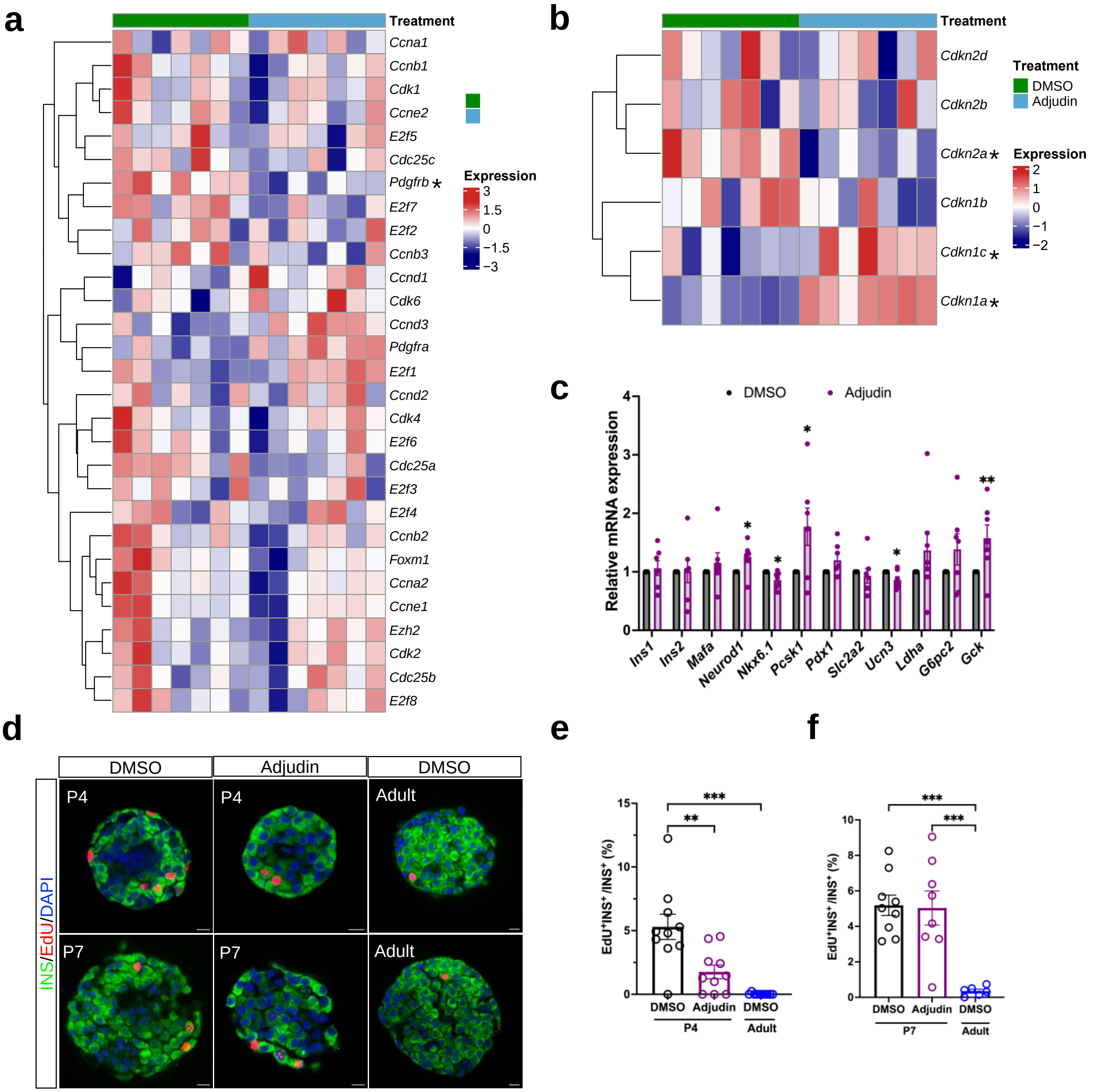

a
Treatment
Ccna1
Ccnb1
Cdk1
Ccne2
E2f5
Cdc25c
Pdgfrb *
E2f7
E2f2
Ccnb3
Ccnd1
Cdk6
Ccnd3
Pdgfra
E2f1
Ccnd2
Cdk4
E2f6
Cdc25a
E2f3
E2f4
Ccnb2
Foxm1
Ccna2
Ccne1
Ezh2
Cdk2
Cdc25b
E2f8
Expression
3
1.5
0
−1.5
−3
b
Treatment
Cdkn2d
Cdkn2b
Cdkn2a *
Cdkn1b
Cdkn1c *
Cdkn1a *
Treatment
DMSO
Adjudin
Expression
2
1
0
−1
−2
c
DMSO
Adjudin
Relative mRNA expression
Ins1
Ins2
Mafa
Neurod1
Nkx6.1
Pcsk1
Pdx1
Slc2a2
Ucn3
Ldha
G6pc2
Gck
d
DMSO
Adjudin
DMSO
P4
P4
Adult
P7
P7
Adult
INS/EdU/DAPI
e
EdU+INS+/INS+ (%)
DMSO
Adjudin
DMSO
P4
Adult
f
EdU+INS+/INS+ (%)
DMSO
Adjudin
DMSO
P7
Adult

ESM Figure 3

ESM Fig. 3 Proliferation effects of Adjudin on P0, P4 and P7 islets. Related to Fig. 2.

(a-b) Heat map of cell cycle activators (a) and cell cycle inhibitors (b) in P0 islets. * indicates significantly regulated genes.

(c) qPCR analysis of the expression of beta cell maturation markers in islets from P4-P7. Islets from P4-P7 islets were cultured in medium containing 11 mmol/l glucose and treated with DMSO or 10 µmol/l Adjudin for 1 day before qPCR. Student's t test. *$p < 0.05$, **$p < 0.01$. n=7 per treatment. Data are presented as mean ± SEM.

(d) Representative immunofluorescence images from whole mount staining of mouse islets to assess proliferation in islets from P4, P7 and adult mice. Islets were cultured in medium containing 11 mmol/l glucose and treated with DMSO or 10 µmol/l Adjudin for 1 day, a final concentration of 20 µmol/l EdU was used for 2 h incubation at the end of the treatment. Scale bar=10 µm.

(e-f) Quantification of percentage of proliferative beta cells in (d). One-way ANOVA with Tukey's multiple-comparisons test. **$p < 0.01$, ***$p < 0.001$. For (e), n=10 per group. For (f), n=9 (P0, DMSO), n=8 (P0, Adjudin), n=6 (adult, DMSO). Data are presented as mean ± SEM.

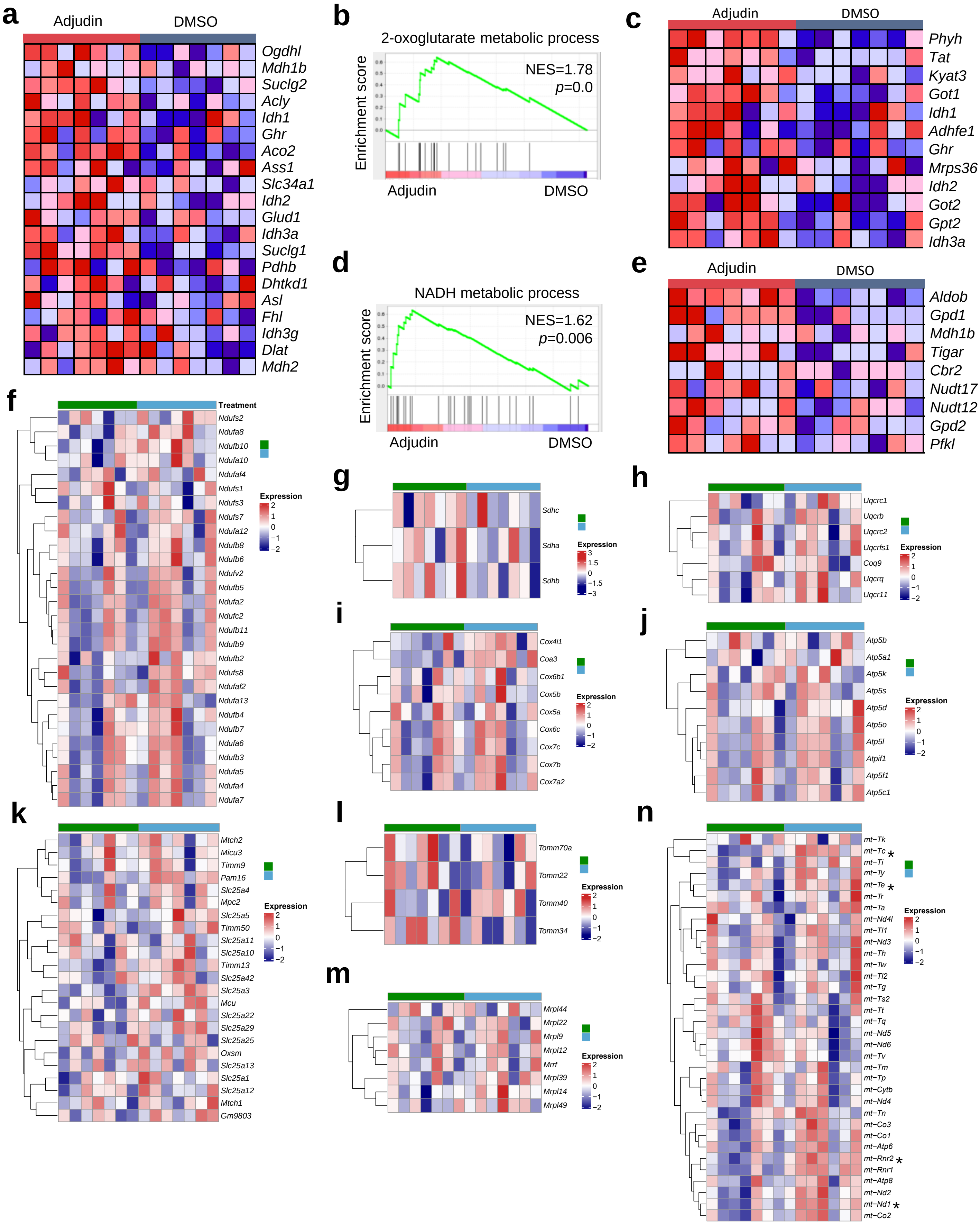

a
Adjudin
DMSO
Ogdhl
Mdh1b
Suclg2
Acly
Idh1
Ghr
Aco2
Ass1
Slc34a1
Idh2
Glud1
Idh3a
Suclg1
Pdhb
Dhtkd1
Asl
Fhl
Idh3g
Dlat
Mdh2
b
2-oxoglutarate metabolic process
Enrichment score
NES=1.78
p=0.0
Adjudin
DMSO
c
Adjudin
DMSO
Phyh
Tat
Kyat3
Got1
Idh1
Adhfe1
Ghr
Mrps36
Idh2
Got2
Gpt2
Idh3a
d
NADH metabolic process
Enrichment score
NES=1.62
p=0.006
Adjudin
DMSO
e
Adjudin
DMSO
Aldob
Gpd1
Mdh1b
Tigar
Cbr2
Nudt17
Nudt12
Gpd2
Pfkl
f
Treatment
Ndufs2
Ndufa8
Ndufb10
Ndufa10
Ndufaf4
Ndufs1
Ndufs3
Ndufs7
Ndufa12
Ndufb8
Ndufb6
Ndufv2
Ndufb5
Ndufa2
Ndufc2
Ndufb11
Ndufb9
Ndufb2
Ndufs8
Ndufaf2
Ndufa13
Ndufb4
Ndufb7
Ndufa6
Ndufb3
Ndufa5
Ndufa4
Ndufa7
Expression
2
1
0
−1
−2
g
Sdhc
Sdha
Sdhb
Expression
3
1.5
0
−1.5
−3
h
Uqcrc1
Uqcrb
Uqcrc2
Uqcrfs1
Coq9
Uqcrq
Uqcr11
Expression
2
1
0
−1
−2
i
Cox4i1
Coa3
Cox6b1
Cox5b
Cox5a
Cox6c
Cox7c
Cox7b
Cox7a2
Expression
2
1
0
−1
−2
j
Atp5b
Atp5a1
Atp5k
Atp5s
Atp5d
Atp5o
Atp5l
Atpif1
Atp5f1
Atp5c1
Expression
2
1
0
−1
−2
k
Mtch2
Micu3
Timm9
Pam16
Slc25a4
Mpc2
Slc25a5
Timm50
Slc25a11
Slc25a10
Timm13
Slc25a42
Slc25a3
Mcu
Slc25a22
Slc25a29
Slc25a25
Oxsm
Slc25a13
Slc25a1
Slc25a12
Mtch1
Gm9803
Expression
2
1
0
−1
−2
l
Tomm70a
Tomm22
Tomm40
Tomm34
Expression
2
1
0
−1
−2
m
Mrpl44
Mrpl22
Mrpl9
Mrpl12
Mrrf
Mrpl39
Mrpl14
Mrpl49
Expression
2
1
0
−1
−2
n
mt−Tk
mt−Tc *
mt−Ti
mt−Ty
mt−Te *
mt−Tr
mt−Ta
mt−Nd4l
mt−Tl1
mt−Nd3
mt−Th
mt−Tw
mt−Tl2
mt−Tg
mt−Ts2
mt−Tt
mt−Tq
mt−Nd5
mt−Nd6
mt−Tv
mt−Tm
mt−Tp
mt−Cytb
mt−Nd4
mt−Tn
mt−Co3
mt−Co1
mt−Atp6
mt−Rnr2 *
mt−Rnr1
mt−Atp8
mt−Nd2
mt−Nd1 *
mt−Co2
Expression
2
1
0
−1
−2

ESM Figure 4

ESM Fig. 4 Mitochondrial signatures of P0 islets. Related to Fig. 2.

(a) Heatmap of the core signature for the gene set of "tricarboxylic acid metabolic process" (Fig.2m), the range of colors (red, pink, light blue, and dark blue) shows the range of expression values (high, moderate, low, and lowest).

(b) GSEA plot showing gene sets related to “2-oxoglutarate metabolic process”. NES, normalized enrichment score.

(c) Heatmap of the core signature for the gene set of "2-oxoglutarate metabolic process" (Fig.2l), the range of colors (red, pink, light blue, and dark blue) shows the range of expression values (high, moderate, low, and lowest).

(d) GSEA plot showing gene sets related to “NADH metabolic process”. NES, normalized enrichment score.

(e) Heatmap of the core signature for the gene set of “NADH metabolic process” (Fig. 2m), the range of colors (red, pink, light blue, and dark blue) shows the range of expression values (high, moderate, low, and lowest).

(f-n) Heatmap showing mitochondrial signatures in P0 islets. (f-j) oxidative phosphorylation complexes I-V. Genes encoding inner (k) and outer (l) mitochondrial membrane transport proteins. (m) genes encoding mitochondrial ribosomal proteins. (n) mitochondrial genes. * indicates significantly regulated genes.

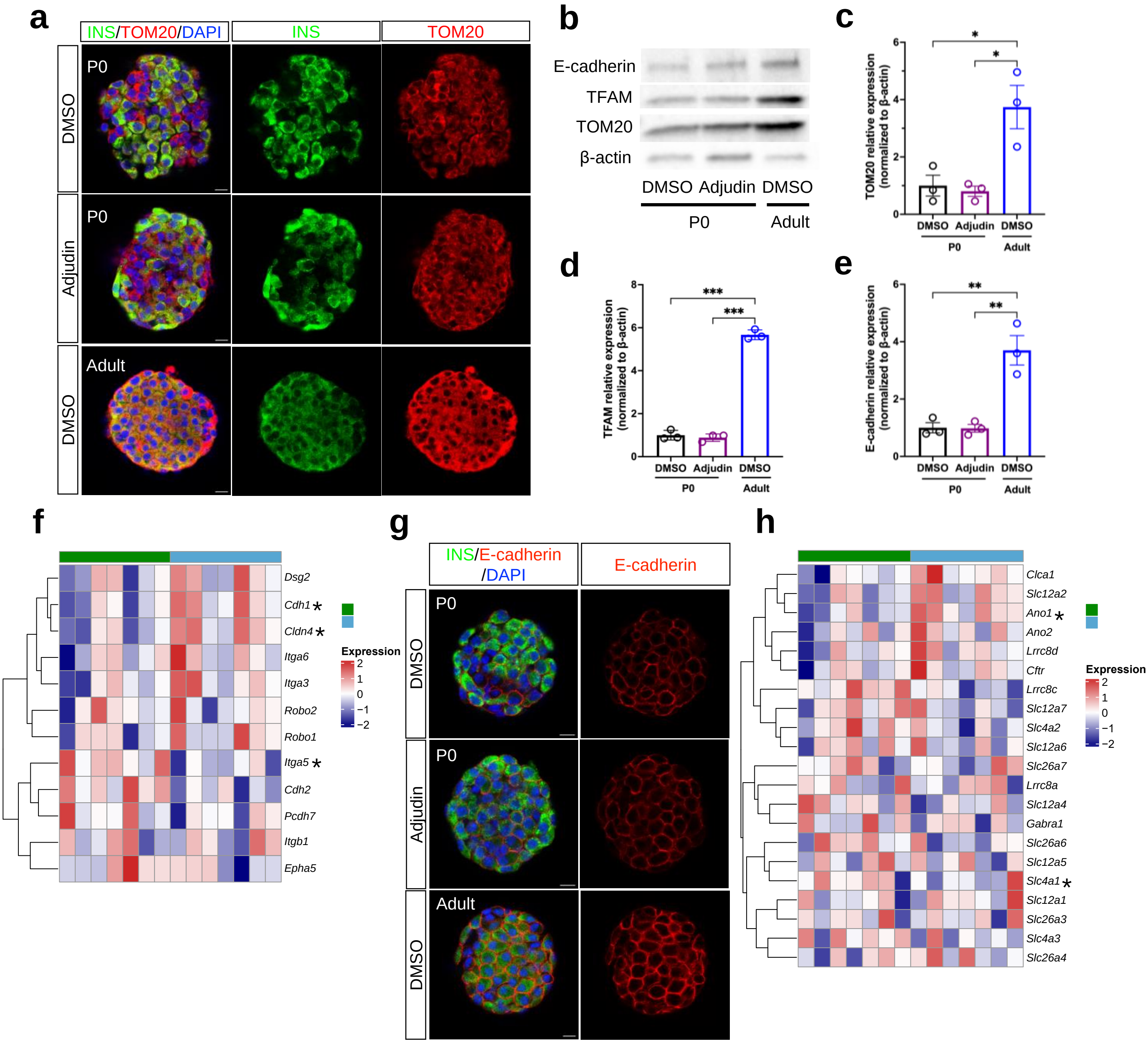
a
INS/TOM20/DAPI
INS
TOM20
DMSO
Adjudin
DMSO
P0
P0
Adult
b
E-cadherin
TFAM
TOM20
β-actin
DMSO Adjudin DMSO
P0
Adult
c
TOM20 relative expression (normalized to β-actin)
DMSO Adjudin DMSO
P0
Adult
d
TFAM relative expression (normalized to β-actin)
e
E-cadherin relative expression (normalized to β-actin)
f
Dsg2
Cdh1 *
Cldn4 *
Itga6
Itga3
Robo2
Robo1
Itga5 *
Cdh2
Pcdh7
Itgb1
Epha5
Expression
g
INS/E-cadherin/DAPI
E-cadherin
h
Clca1
Slc12a2
Ano1 *
Ano2
Lrrc8d
Cftr
Lrrc8c
Slc12a7
Slc4a2
Slc12a6
Slc26a7
Lrrc8a
Slc12a4
Gabra1
Slc26a6
Slc12a5
Slc4a1 *
Slc12a1
Slc26a3
Slc4a3
Slc26a4

ESM Figure 5

ESM Fig. 5 Effects of Adjudin on mitochondria and cell-cell contact in P0 islets. Related to Fig. 2.

(a) Representative immunofluorescence images from whole mount staining of mouse islets to assess expression of TOM20. Islets were cultured in medium containing 11 mmol/l glucose and treated with DMSO or 10 μmol/l Adjudin for 1 day before the fixation and whole mount staining. Scale bar=10 μm.

(b) Representative image from western blot for TOM20, TFAM, E-caherin and beta-actin in mouse islets. Islets were cultured in medium containing 11 mmol/l glucose and treated with DMSO or 10 μmol/l Adjudin for 1 day before western blot.

(c-e) Quantification of western blot bands in (b), each dot represent data from one biological sample. One-way ANOVA with Tukey's multiple-comparisons test. $*p < 0.05$, $**p < 0.01$, $***p < 0.001$. n=3 per treatment. Data are presented as mean ± SEM.

(f) Heat map of cell-cell contact genes in P0 islets. * indicates significantly regulated genes.

(g) Representative immunofluorescence images from whole mount staining of mouse islets to assess expression of E-cadherin. Islets were cultured in medium containing 11 mmol/l glucose and treated with DMSO or 10 μmol/l Adjudin for 1 day before the fixation and whole mount staining. Scale bar=10 μm.

(h) Heat map of genes associated with chloride channels. * indicates significantly regulated genes.

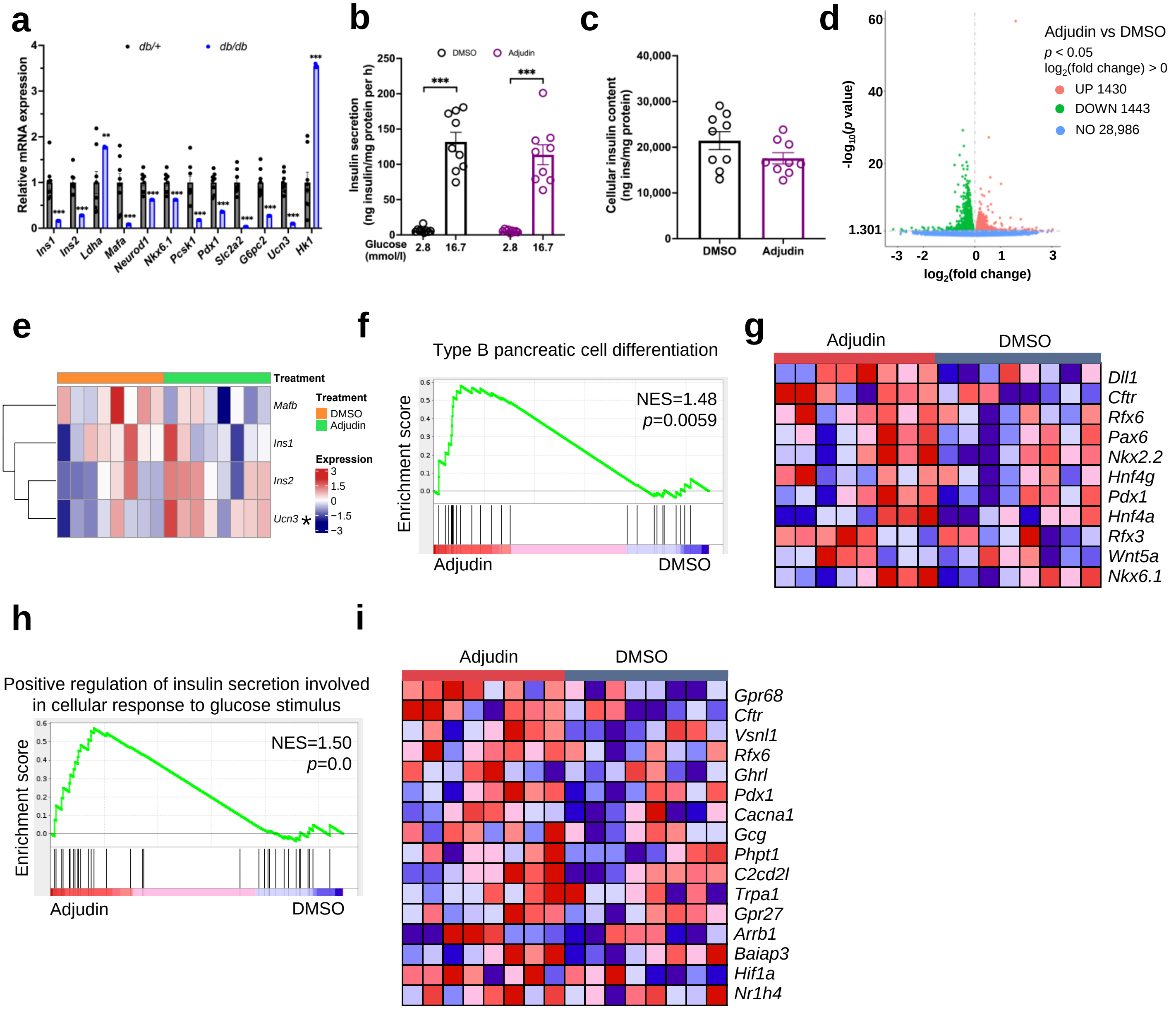
a
b
c
d
e
f
g
h
i
Relative mRNA expression
db/+
db/db
Ins1
Ins2
Ldha
Mafa
Neurod1
Nkx6.1
Pcsk1
Pdx1
Slc2a2
G6pc2
Ucn3
Hk1
Insulin secretion (ng insulin/mg protein per h)
DMSO
Adjudin
Glucose (mmol/l)
2.8
16.7
Cellular insulin content (ng ins/mg protein)
Adjudin vs DMSO
p < 0.05
log2(fold change) > 0
UP 1430
DOWN 1443
NO 28,986
-log10(p value)
log2(fold change)
Treatment
Expression
Type B pancreatic cell differentiation
Enrichment score
NES=1.48
p=0.0059
Dll1
Cftr
Rfx6
Pax6
Nkx2.2
Hnf4g
Pdx1
Hnf4a
Rfx3
Wnt5a
Nkx6.1
Positive regulation of insulin secretion involved in cellular response to glucose stimulus
NES=1.50
p=0.0
Gpr68
Cftr
Vsnl1
Rfx6
Ghrl
Pdx1
Cacna1
Gcg
Phpt1
C2cd2l
Trpa1
Gpr27
Arrb1
Baiap3
Hif1a
Nr1h4

ESM Figure 6

ESM Fig. 6 Effects of Adjudin on *db/db* islets. Related to Fig. 3.

(a) qPCR analysis of the expression of beta cell maturation markers in *db/db* and *db/+* islets. Student's t test. $^{**}p < 0.01$, $^{***}p < 0.001$. n=8 (*db/+*), n=10 (*db/db*). Data are presented as mean ± SEM.

(b) GSIS of *db/db* islets. Islets were cultured in medium containing 11 mmol/l glucose and treated with DMSO or 10 µmol/l Adjudin for 1 day before the GSIS. Student's t test. $^{***}p < 0.001$. n=9 per treatment. Data are presented as mean ± SEM.

(c) Insulin content in *db/db* islets. Islets were cultured in medium containing 11 mmol/l glucose and treated with DMSO or 10 µmol/l Adjudin for 1 day before GSIS, and islet insulin content was measured at the end of the GSIS. Student's t test. n=9 per treatment. Data are presented as mean ± SEM.

(d) Volcano plot for *db/db* islets.

(e) Heatmap of beta cell maturation markers in *db/db* islets. * indicates significantly regulated genes.

(f) GSEA plot showing gene sets related to "type B pancreatic cell differentiation" in *db/db* islets. NES, normalized enrichment score.

(g) Heatmap of the core signature for the gene set of "type B pancreatic cell differentiation" in (f), the range of colors (red, pink, light blue, and dark blue) shows the range of expression values (high, moderate, low, and lowest).

(h) GSEA plot showing gene sets related to "positive regulation of insulin secretion involved in cellular response to glucose stimulus" in *db/db* islets. NES, normalized enrichment score.

(i) Heatmap of the core signature for the gene set of "positive regulation of insulin secretion involved in cellular response to glucose stimulus" in (h), the range of colors (red, pink, light blue, and dark blue) shows the range of expression values (high, moderate, low, and lowest).

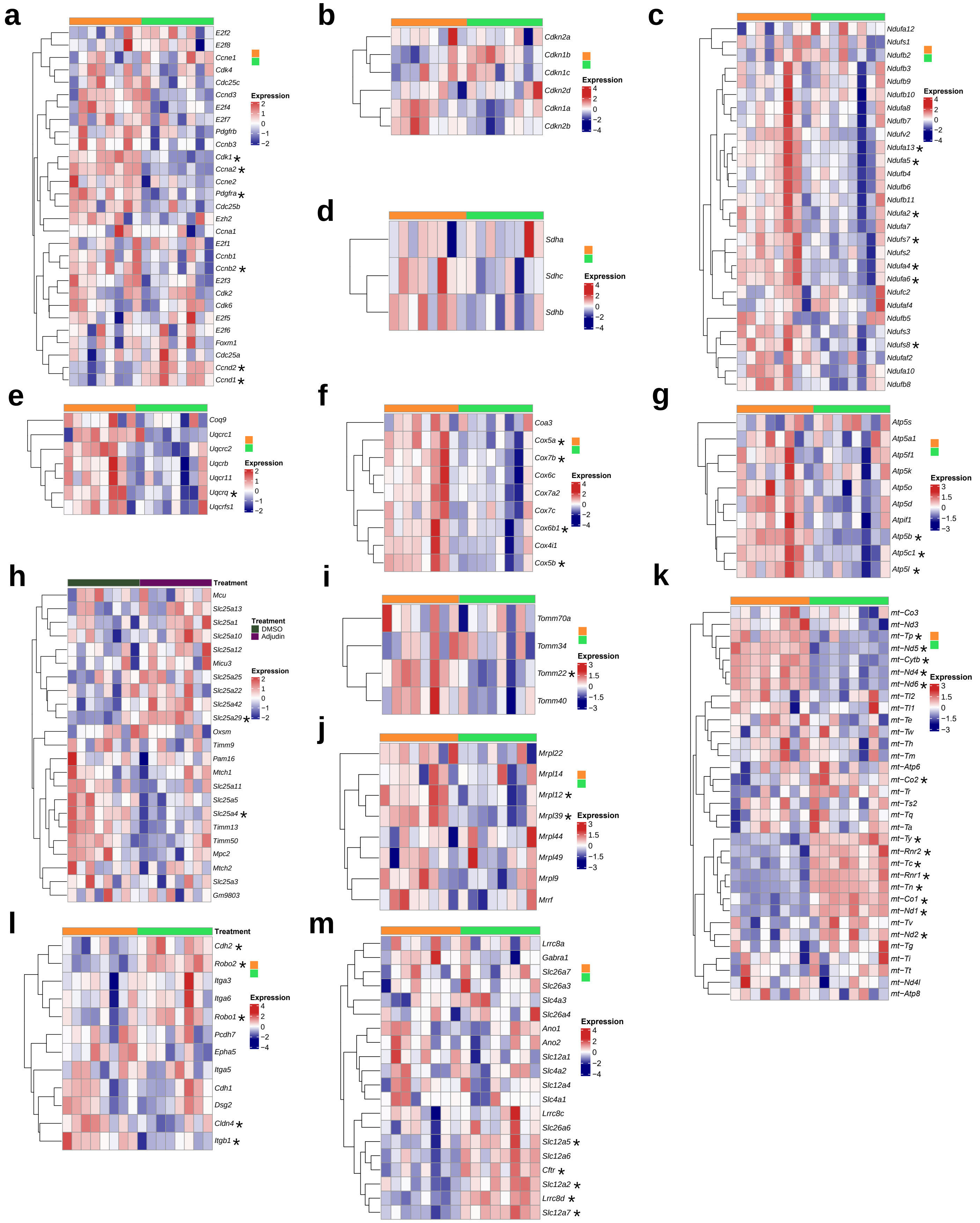
a
E2f2
E2f8
Ccne1
Cdk4
Cdc25c
Ccnd3
E2f4
E2f7
Pdgfrb
Ccnb3
Cdk1 *
Ccna2 *
Ccne2
Pdgfra *
Cdc25b
Ezh2
Ccna1
E2f1
Ccnb1
Ccnb2 *
E2f3
Cdk2
Cdk6
E2f5
E2f6
Foxm1
Cdc25a
Ccnd2 *
Ccnd1 *
Expression
b
Cdkn2a
Cdkn1b
Cdkn1c
Cdkn2d
Cdkn1a
Cdkn2b
Expression
c
Ndufa12
Ndufs1
Ndufb2
Ndufb3
Ndufb9
Ndufb10
Ndufa8
Ndufb7
Ndufv2
Ndufa13 *
Ndufa5 *
Ndufb4
Ndufb6
Ndufb11
Ndufa2 *
Ndufa7
Ndufs7 *
Ndufs2
Ndufa4 *
Ndufa6 *
Ndufc2
Ndufaf4
Ndufb5
Ndufs3
Ndufs8 *
Ndufaf2
Ndufa10
Ndufb8
Expression
d
Sdha
Sdhc
Sdhb
Expression
e
Coq9
Uqcrc1
Uqcrc2
Uqcrb
Uqcr11
Uqcrq *
Uqcrfs1
Expression
f
Coa3
Cox5a *
Cox7b *
Cox6c
Cox7a2
Cox7c
Cox6b1 *
Cox4i1
Cox5b *
Expression
g
Atp5s
Atp5a1
Atp5f1
Atp5k
Atp5o
Atp5d
Atpif1
Atp5b *
Atp5c1 *
Atp5l *
Expression
h
Treatment
DMSO
Adjudin
Mcu
Slc25a13
Slc25a1
Slc25a10
Slc25a12
Micu3
Slc25a25
Slc25a22
Slc25a42
Slc25a29 *
Oxsm
Timm9
Pam16
Mtch1
Slc25a11
Slc25a5
Slc25a4 *
Timm13
Timm50
Mpc2
Mtch2
Slc25a3
Gm9803
Expression
i
Tomm70a
Tomm34
Tomm22 *
Tomm40
Expression
j
Mrpl22
Mrpl14
Mrpl12 *
Mrpl39 *
Mrpl44
Mrpl49
Mrpl9
Mrrf
Expression
k
mt−Co3
mt−Nd3
mt−Tp *
mt−Nd5 *
mt−Cytb *
mt−Nd4 *
mt−Nd6 *
mt−Tl2
mt−Tl1
mt−Te
mt−Tw
mt−Th
mt−Tm
mt−Atp6
mt−Co2 *
mt−Tr
mt−Ts2
mt−Tq
mt−Ta
mt−Ty *
mt−Rnr2 *
mt−Tc *
mt−Rnr1 *
mt−Tn *
mt−Co1 *
mt−Nd1 *
mt−Tv
mt−Nd2 *
mt−Tg
mt−Ti
mt−Tt
mt−Nd4l
mt−Atp8
Expression
l
Treatment
Cdh2 *
Robo2 *
Itga3
Itga6
Robo1 *
Pcdh7
Epha5
Itga5
Cdh1
Dsg2
Cldn4 *
Itgb1 *
Expression
m
Lrrc8a
Gabra1
Slc26a7
Slc26a3
Slc4a3
Slc26a4
Ano1
Ano2
Slc12a1
Slc4a2
Slc12a4
Slc4a1
Lrrc8c
Slc26a6
Slc12a5 *
Slc12a6
Cftr *
Slc12a2 *
Lrrc8d *
Slc12a7 *
Expression

ESM Figure 7

ESM Fig. 7 Heat maps for *db/db* islets. Related to Fig.3.

Heatmaps of expression changes in *db/db* islet with or without Adjudin treatment in genes related to cell cycle activator (a) and inhibitor (b), oxidative phosphorylation complexes I-V (c-g), inner (h) and outer (i) mitochondrial membrane transport, mitochondrial ribosomal proteins (j), mitochondrial genes (k), cell-cell contact (l) and chloride channels (m). * indicates significantly regulated genes.

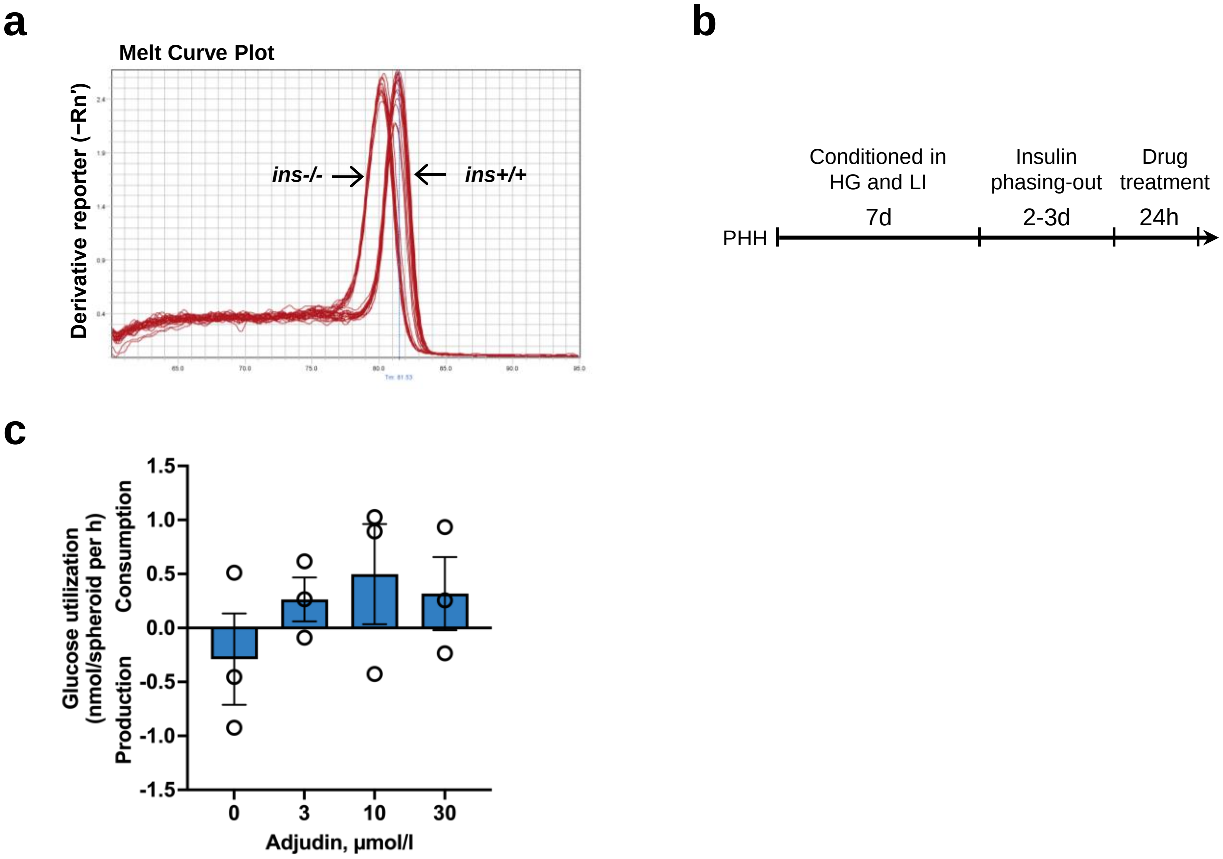
a
Melt Curve Plot
Derivative reporter (−Rn')
ins-/-
ins+/+
b
Conditioned in HG and LI
Insulin phasing-out
Drug treatment
PHH
7d
2-3d
24h
c
Glucose utilization (nmol/spheroid per h)
Consumption
Production
1.5
1.0
0.5
0.0
-0.5
-1.0
-1.5
0
3
10
30
Adjudin, µmol/l

ESM Figure 8

ESM Fig. 8 Genotyping results of insulin mutant fish and insulin-independent effects of Adjudin on human liver spheroids. Related to Fig. 4.

(a) Melt curve analysis of qPCR for genotyping larvae that were *ins+/+* or *ins-/-*.

(b) Schematic of how human hepatocytes were conditioned in low physiological insulin levels (LI) and high physiological glucose levels (HG) for 7 days, followed by phasing out insulin, and treatment with DMSO, 3 µmol/l, 10 µmol/l or 30 µmol/l Adjudin for 24 hours. The glucose level in the medium was measured before and after compound treatment.

(c) Glucose production/consumption in the PHH spheroids treated as described in (b). One-way ANOVA followed by Dunnett's multiple comparisons test. n=3 independent biological replicates per treatment. Data are presented as mean ± SEM.

a

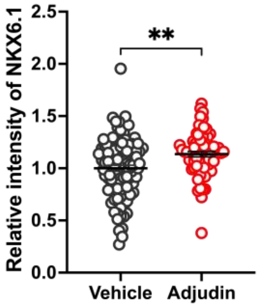

b

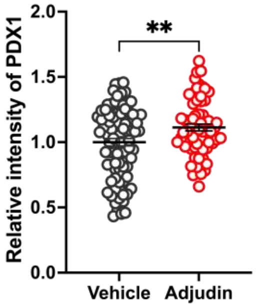

ESM Figure 9

ESM Fig. 9 Effects of Adjudin on beta cells in *db/db* mouse. Related to Fig. 5.

(a) Quantification of relative intensity of NKX6.1 staining per islet. Student’s t test. $**p < 0.01$. n=101 islets (vehicle). n= 67 islets (Adjudin). Data are presented as mean ± SEM.

(n) Quantification of relative intensity of PDX1 staining per islet. Student’s t test. $**p < 0.01$. n=90 islets (vehicle). n= 65 islets (Adjudin). Data are presented as mean ± SEM.

ESM Video 1. Live calcium imaging of beta cells in a DMSO-treated larva after 1 day of beta cell regeneration. Related to Fig. 1f and 1h.

An example video of live calcium imaging of beta cells in a *Tg(ins:GCaMP6s);Tg(ins:H2BmCherry);Tg(ins:Flag-NTR)* zebrafish larva at 5 dpf after 1 day of beta cell regeneration, with beta cells expressing H2BmCherry shown in red, calcium signal in green. The zebrafish larvae were treated with DMSO for 1 day after beta cell ablation from 3-4 dpf, and had calcium signal recorded at 5 dpf.

ESM Video 2. Live calcium imaging of beta cells in a Adjudin-treated larva after 1 day of beta cell regeneration. Related to Fig. 1g and 1i.

An example video of live calcium imaging of beta cells in a *Tg(ins:GCaMP6s);Tg(ins:H2BmCherry);Tg(ins:Flag-NTR)* zebrafish larva at 5 dpf after 1 day of beta cell regeneration, with beta cells expressing H2BmCherry shown in red, calcium signal in green. The zebrafish larvae were treated with Adjudin for 1 day after beta cell ablation from 3-4 dpf, and had calcium signal recorded at 5 dpf.

ESM Video 3. Live calcium imaging of beta cells in a larva subjected to acute treatment with Adjudin. Related to ESM Fig. 1.

An example video of live calcium imaging of beta cells in a *Tg(ins:GCaMP6s);Tg(ins:H2BmCherry)* fish at 5 dpf, with beta cells expressing H2BmCherry shown in red, calcium signal in green. The zebrafish larvae were treated with 5% glucose from 3-4 dpf, then glucose was washed out, and calcium signal was recorded at 5 dpf before and after acute treatment with Adjudin.
